# The crosslinks between ferroptosis and autophagy in asthma

**DOI:** 10.3389/fimmu.2023.1140791

**Published:** 2023-03-29

**Authors:** Xiaodi Lv, Weifeng Tang, Jingjing Qin, Wenqian Wang, Jingcheng Dong, Ying Wei

**Affiliations:** ^1^ Department of Integrative Medicine, Huashan Hospital, Fudan University, Shanghai, China; ^2^ Institutes of Integrative Medicine, Fudan University, Shanghai, China

**Keywords:** asthma, autophagy, crosslinks, ferroptosis, targets

## Abstract

Autophagy is an evolutionarily conserved cellular process capable of degrading various biological molecules and organelles *via* the lysosomal pathway. Ferroptosis is a type of oxidative stress-dependent regulated cell death associated with the iron accumulation and lipid peroxidation. The crosslinks between ferroptosis and autophagy have been focused on since the dependence of ferroptosis on autophagy was discovered. Although the research and theories on the relationship between autophagy and ferroptosis remain scattered and fragmented, the crosslinks between these two forms of regulated cell death are closely related to the treatment of various diseases. Thereof, asthma as a chronic inflammatory disease has a tight connection with the occurrence of ferroptosis and autophagy since the crosslinked signal pathways may be the crucial regulators or exactly regulated by cells and secretion in the immune system. In addition, non-immune cells associated with asthma are also closely related to autophagy and ferroptosis. Further studies of cross-linking asthma inflammation with crosslinked signaling pathways may provide us with several key molecules that regulate asthma through specific regulators. The crosslinks between autophagy and ferroptosis provide us with a new perspective to interpret and understand the manifestations of asthma, potential drug discovery targets, and new therapeutic options to effectively intervene in the imbalance caused by abnormal inflammation in asthma. Herein, we introduce the main molecular mechanisms of ferroptosis, autophagy, and asthma, describe the role of crosslinks between ferroptosis and autophagy in asthma based on their common regulatory cells or molecules, and discuss potential drug discovery targets and therapeutic applications in the context of immunomodulatory and symptom alleviation.

## 1 Introduction

The scientific observation of regulated cell death (RCD) historically originated in 1842 when dying cells in toads were discovered by Karl Vogt. And when the term “apoptosis” was coined in 1972 by Andrew Wyllie, Alastair Currie1, and John Kerr, the surge in RCD research started. Since then, various types of forms of RCD have been explored in the context of the role in multiple pathological and physiological processes of different diseases, the macroscopic morphological alterations, the molecular mechanisms and signaling pathways associated with activation or regulation, and the program in response to varieties of stresses, especially oxidative stress. With multiple novel forms of RCD identified, the core and specific molecular mechanisms have been discovered to insulate the particular form of RCD from others. The research on the regulation of these forms of RCD provided us with novel targets for treating a wide range of diseases. However, it has been increasingly apparent that these molecular programs are deeply interwoven. Studies probing cell death crosstalk have demonstrated multiple molecular interactions between signal transduction pathways and shown that numerous cell death programs are involved in the pathogenesis and pathophysiological process of various diseases. For example, the pyroptotic molecules can activate apoptotic substrates and vice versa, while inhibition of one type of cell death pathway by the pathogen or other signaling defects can result in another pathway of RCD compensating (1–4).

**Figure 1:**
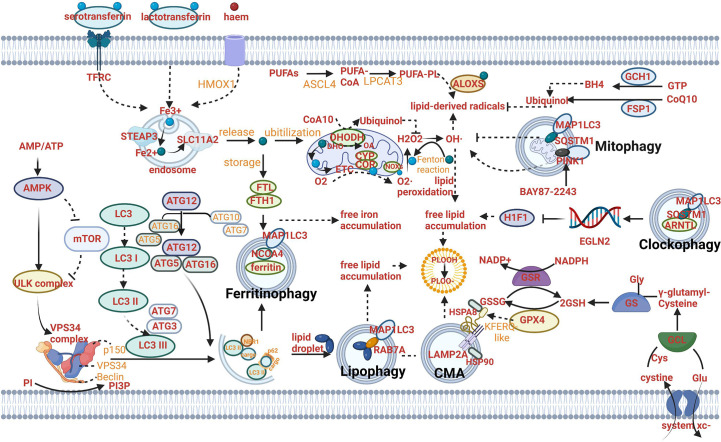
Mechanism of autophagy-dependent ferroptosis. **(A)** The mechanism of autophagy: AMPK can activate ULK complex to induce VPS34 complex and suppress the activity of mTOR and ULK complex. The autophagosome (AP) formation depends on LC3 lipidation that LC3 is cleaved into the soluble form LC3I acting as a precursor to LC3II. These receptors play a central role in the selective recruitment of specific cargoes with ubiquitin labeling during autophagy. **(B)** The mechanism of ferritinophagy: Fe^2+^ can be exported as ferritin through exosomes. NCOA4-mediated ferritinophagy (namely, the autophagic degradation of ferritin) promotes ferroptosis by increasing intracellular iron (Fe^2+^) levels. **(C)** The mechanism of lipophagy: Lipophagy (namely, the autophagic degradation of lipid droplets) increases the levels of free fatty acids available for subsequent lipid peroxidation during ferroptosis. **(D)** The mechanism of CMA: Chaperone-mediated autophagy is involved in GPX4 degradation for ferroptosis. **(E)** The mechanism of mitophagy: Mitophagy (the autophagic degradation of mitochondria) has a dual role in ferroptosis. **(F)** The mechanism of clockophagy: Sequestosome 1-mediated degradation of ARNTL by autophagy (a process termed clockophagy) regulates HIF1α, facilitating ferroptosis. **(G)** The mechanism of ferroptosis: The cystine/glutamate transporter (also known as system xc^−^) imports cystine into cells with a 1:1 counter-transport of glutamate. Once in cells, cystine (Cys_2_) can be oxidized to cysteine (Cys), which is used to synthesize glutathione (GSH) in a reaction catalysed by glutamate–cysteine ligase (GCL) and glutathione synthetase (GSS). By using GSH as a reducing cofactor, glutathione peroxidase GPX4 is capable of reducing lipid hydroperoxides to lipid alcohols. The GSH–GPX4 antioxidation system has an important role in protecting cells from ferroptosis. The AIFM2–CoQ10, ESCRT-III membrane repair and GCH1–BH_4_ systems can also inhibit ferroptosis. Several proteins (including serotransferrin, transferrin receptor (TFRC), solute carrier family 40 member 1 (SLC40A1), ferritin components (FTH1 and FTL), nuclear receptor co-activator 4 (NCOA4) and prominin 2) control ferroptosis through the regulation of iron metabolism. Acetyl-CoA carboxylase (ACAC)-mediated fatty acid synthesis or lipophagy-mediated fatty acid release induces the accumulation of intracellular free fatty acids, which fuels ferroptosis. Long-chain fatty acid–CoA ligase 4 (ACSL4) and lysophospholipid acyltransferase 5 (LPCAT3) promote the incorporation of polyunsaturated fatty acids (PUFAs) into phospholipids to form polyunsaturated fatty acid-containing phospholipids (PUFA–PLs), which are vulnerable to free radical-initiated oxidation mediated by lipoxygenases (ALOXs).

The crosslinks between ferroptosis and macroautophagy/autophagy have been focused on since the form of autophagy-dependent ferroptosis was discovered. The initiation of autophagy is mediated by the unc-51-like kinase (ULK) complex, which can be inhibited by mTOR complex 1 (mTORC1) or activated by 5′-AMP-activated protein kinase (AMPK) (the kinase can be activated by stress signals). Then, PI3P-binding molecules can be recruited after the activation of vacuolar protein sorting 34 (VPS34) to form a phagosome (an isolated pre-autophagosomal structure) (5–8). The autophagosome (AP) formation depends on LC3 lipidation that LC3 is cleaved into the soluble form LC3I acting as a precursor to LC3II, a docking point covalently attaching to the phagosome membrane for cargo receptors (**Figure 1A**) (9–11). These receptors play a central role in the selective recruitment of specific cargoes with ubiquitin labeling during autophagy (**Figure 1A**). Then, an AP can be formed when the phagosome extends and eventually closes (**Figure 1A**). When transported to the perinuclear region and fused with proximal lysosomes, cargoes can be degraded and nutrients can be recycled through lysosomal hydrolases, along with the formation of autolysosomes (ALs) (12–15). Ferroptosis is initiated with lipid peroxidation which is uncontrolled and lethal resulting in subsequent rupture of the plasma membrane through iron catalysis, containing enzymatic (lipoxygenases) and non-enzymatic (Fenton’s reaction) mechanisms. Iron accumulation and lipid peroxidation are two critical mediators in ferroptosis. Autophagy can modulate ferroptosis based on particular lysosomal degradation of specific organelles or proteins leading to iron accumulation and lipid peroxidation. Similarly, ferroptosis can regulate the formation of ALs to affect the process of autophagy. For example, ferroptosis induction has been proven to have a tight connection with the turnover of lipidated LC3 and the fusion of the AP with lysosomes (16, 17).

Furthermore, the signal transduction pathways or principal signal molecules of two forms of RCD may be common or cross-linked. The research on these crosslinked pathways and molecules can provide more effective pharmacological targets for improving the prognosis of multiple patients. In addition, both ferroptosis and autophagy have been proven to have a tight connection with innate and adaptive immunity. On the one hand, the activation of immune cells can be regulated by these two forms of RCD. On the other hand, the immune and inflammatory signaling can be controlled by the pathways or proteins of ferroptosis and autophagy.

Asthma is a heterogeneous and complex disorder characterized by asthmatic inflammation in the airways. In addition to the chronic inflammation, airway remodeling, and bronchial hyper-reactivity comprise the specific pathogenesis of asthma along with the interaction of non-immune cells such as epithelial cells and airway smooth muscle (ASM) cells, and immune cells including the cells from the innate and adaptive immune systems. Immune factors and various genetic factors interplay in an array of disorders after being stimulated by different environmental factors. Asthma attacks occur over periods of many years, which creates additional therapeutic challenges. Long term structural airway alteration involves multiple cell types and leads to non-reversible obstruction of airflow causing chronic symptoms and, in rare cases, death. New targets for asthmatic therapy have been always discovered based on various areas of lung research to circumvent some of the current limitations of conventional asthma therapy that include tachyphylaxis to beta adrenergic agonists, corticosteroid insensitivity, off-target effects of corticosteroids, and improvement of effective treatments to reverse obstructive airway remodeling.

Autophagy and ferroptosis as two crucial forms of RCD have been proved to enrich the strategies of asthmatic therapy. For example, a randomized clinical trial demonstrated that Carbamazepine, an anticonvulsant drug and autophagy inducer had high efficacy in therapy of moderate or severe bronchial asthma (18). Carbamazepine has been shown to induce antimicrobial autophagy through mTOR-independent pathway, suggesting that autophagy induction by repurposed drug could provide an easily implementable potential therapy for some asthma phenotypes (19). Similarly, ferroptosis was reported to have a tight connection with type 2 high asthma. The elevated expression of ALOX15 (arachidonate 15- lipoxygenase), a key enzyme of lipid peroxidation, in the bronchial epithelium or eosinophils of BALF in both childhood and adult asthmatics is associated with allergen sensitization and airway inflammation (20, 21). Furthermore, Phosphatidylethanolamine binding protein 1 (PEBP1), the crosslinked regulatory molecule of ferroptosis and autophagy, has been discovered to affect the function and survival of asthmatic epithelial cells. PEBP1 which is also called rheostat between ferroptosis and autophagy in HAECs can interact with ALOX15 to induce ferroptosis in asthmatic HAECs by generating 15-hydroperoxyeicosaetetranoic acid (15-HpETE-PE). In addition, when the process of PEBP1 binding with LC3-I is inhibited, autophagic pro-survival pathways would be activated in asthmatic HAECs and subsequently, cell destruction would be limited (22).

However, the role of ferroptosis and autophagy in asthma is mostly preclinical evidence, a series of evaluation criteria should be developed before clinical application. As such a large player in general function of cells associated with asthma, autophagy and ferroptosis do provide multiple therapeutic targets. Determining the roles they play in different cell types is key to understanding how to specifically target them. Moreover, in order to reach the best clinical outcome, it is also crucial to consider the stage of development of the disease. Indeed, depending if asthmatic patients are in the initiation or exacerbation phases of the pathogenesis, the specific cell type to be targeted should be considered.

It can be further concluded that the therapeutic schedules to attenuate the symptoms and improve the prognosis for asthma patients can be established based on regulating the innate and adaptive immunity *via* modulating the progress of autophagy and ferroptosis, or discovering some crosslinked targets of ferroptosis and autophagy in various cells which are crucial in asthmatic pathogenesis. All in all, the crosslinks between ferroptosis and autophagy may provide us with more effective targets in various specific cell types to treat asthma.

CD11b^+^ cDCs can maturate and migrate depending on the transcription factor IRF4 with the effect of danger signals and ‘instructive’ cytokines produced by HAECs. The typical function of DCs as antigen-presenting cells is to internalize antigens and present antigen-derived peptides to T cells. When activated by the innate immune system, CD4^+^T cells can differentiate into multiple functional subsets of helper T cells such as Th2 cells, Th17 cells, Th1 cells, and Treg cells. Both Th2 cells and ILC2 cells contribute to eosinophilic inflammation by upregulating the expression of GATA-3 which can promote the production of Th2 cytokines, and increase the production of IL-5 to modulate the development of eosinophils, IL-13 leading to goblet cell metaplasia and bronchial hyperreactivity, IL-4 affecting the mature and activation of Th9 cells which can promote the IgE synthesis by B cells. Both Th1 and Th17 cells can result in neutrophilic inflammation by respectively secreting IFN-γ or TNFαand IL-17A or IL-17F Treg cells, a subset of CD4^+^ T cells, also originate from Th0 cells. The fate of follicular helper T cells (TFH) can be adopted by Th cells producing IL-21 so that IgE can be induced by B lymphocytes.

## 2 The role of autophagy in ferroptosis

Ferroptosis is promoted by iron accumulation and lipid peroxidation. The progress of both initiation and advancement of ferroptosis involves autophagy. The process of autophagy can be divided into non-selective autophagy and selective autophagy. Non-selective autophagy has long been thought to be the main form of bulk degradation pathway, which randomly engulfs a portion of the cytoplasm into autophagosomes and then delivers them to lysosome for degradation. Selective autophagy, however, specifically recognizes and degrades the particular cargo, either a protein complex, an organelle, or lipid droplets (23). Nonselective autophagy is primarily a starvation response, whereas cells use selective autophagy for a variety of purposes, such as remodeling to adapt to changing environmental/nutritional conditions and to eliminate damaged organelles.

### 2.1 The role of selective autophagy in ferroptosis

The selective autophagy processes which influence ferroptosis included two main parts in the context of regulating the level of iron and modulating lipid peroxidation.

#### 2.1.1 The role of selective autophagy in regulating the level of iron

The level of iron can be regulated by ferritinophagy mediated by nuclear receptor coactivator 4 (NCOA4) (**Figure 1B**) (16, 17). The role of ferritinophagy in iron accumulation promotes the development of ferroptosis. Hence, the suppression of ferritinophagy can increase iron storage and limit ferroptosis by modulating the genetic expression of LC3, autophagy-related gene (ATG)3, ATG5, ATG7, ATG13 or ELAV-like RNA-binding protein 1 (ELAVL1/HuR) (24, 25). Furthermore, the strategy of suppressing ferroptosis from the perspective of upregulating iron storage can be achieved by poly (RC)- binding proteins (PCBPs) and ferritin mitochondrial (FTMT). PCBPs acting as iron chaperones can deliver Fe2^+^ to ferritin, thereby limiting ferroptosis (26). Similarly, when the principal iron storage protein in mitochondria, FTMT, is upregulated, ferroptosis induced by erastin can be inhibited (27).

#### 2.1.2 The role of selective autophagy in modulating lipid peroxidation

The selective autophagy plays a crucial role in modulating lipid peroxidation. Thereof, lipophagy and clockophagy have a tight relationship with free lipid accumulation. Mitophagy and chaperon-mediated autophagy (CMA) are respectively responsible for impaired oxidative phosphorylation and lipid ROS accumulation.

Lipophagy can decompose lipid droplets (LDs) which are necessary for cells to resist oxidative stress. Lipid peroxidation has been proved to be induced by polyunsaturated fatty acids (PUFAs) which can be transported into their center along with the formation of LDs (**Figure 1C**) (28).

Hence, when lipophagy is initiated and enhanced, lipid peroxidation can be triggered due to the increased PUFAs and result in subsequent ferroptosis. Furthermore, ferroptosis promoted by lipophagy can be suppressed after the knockdown of Rab7a member RAS oncogene family (RAB7A) *in vitro* (29).

Compared to the direct relation between lipophagy and free lipid accumulation, the mechanism of clockophagy regulating ferroptosis presents complex. Aryl hydrocarbon receptor nuclear translocator-like (ARNTL/BMAL1) as the center circadian clock protein can be degraded by clockophagy and result in negative modulation of the transcription factor hypoxia-inducible factor 1(HIF1) through transcriptionally upregulating the expression of Egl-9 family HIF2, which is responsible for upregulating expression of specific genes involved in regulating the transport and combination of fatty acids and lipids (for example, FABP3 and FABP7). When HIF1 is deficient, downregulation of these proteins produced by the aforementioned specific genes can stimulate ferroptosis by preventing the combination of lipids and fatty acids and their transportation from the plasma membrane to mitochondria, and promoting their peroxidation (30). Sequestosome 1 (SQSTM1/P62) as the autophagy receptor for clockophagy mediates ARNTL degradation and promotes ferroptosis (**Figure 1F**) (30).

The effect of CMA on lipid peroxidation centers on the function of degradation of the antioxidant defense systems. In addition, CMA can provide a pathway to degrade various proteins from the cytoplasm in lysosomes directly (31). All pathways begin with a combination of heat shock protein family A (Hsp70) member 8 (HSPA8/HSC70) and proteins with a KFERQ-like motif (**Figure 1D**). Then the lysosomes can degrade these specific proteins with the particular motif through the recognition of the lysosome-associated membrane protein type 2A (LAMP2A). A key enzyme of the antioxidant defense systems called glutathione peroxidase 4 (GPX4) in ferroptosis is a protein containing KEFRQ-like motif. It has been proven to be degraded by HSP90-mediated CMA during erastin-induced ferroptosis. 2-amino-5-chloro-N,3-dimethylbenzamide (CDDO) can block the combination between HSP90 and LAMP2A. Therefore, when erastin-induced ferroptosis occurs, CDDO can suppress the progress by preventing the degradation of GPX4 mediated by CMA (32). GPX4 plays a principal role in maintaining the intracellular antioxidant environment according to reducing phospholipid hydroperoxide production (AA/AdA-PE-OOH) (which is the immediate cause of lipid peroxidation and ferroptosis) to the corresponding phospholipid alcohol (PLOH). The activity or expression of GPX4 is affected by GSH and selenium (33, 34). When GPX is synthesized, selenium can replace the sulfur of cysteine (amino acid of an emerging polypeptide chain) and involve in the generation of selenocysteine (Sec) due to the stop codon UGA “recoded” by a selenocysteine insertion sequence (SECIS). The anti-ferroptotic activity of GPX4 can be enhanced through a selenocysteine residue at 46 (U46) (33). In the catalytic cycle of GPX4, GSH can reduce the selenic acid (-SeOH) to the intermediate selenide disulfide (-Se-SG). Finally, the second GSH can activate GPX4, and glutathione disulfide (GS-SG) can be released (**Figure 1G**). The synthetic reaction of GSH originates from system xc^-^, the cystine/glutamate transporter (**Figure 1G**). The transporter can import cystine into cells and meanwhile transport the same quantities of glutamate out of cells. System xc^-^ is composed of SLC3A2 and SLC7A11 (**Figure 1G**). Like CMA degrading GPX4, the whole antioxidant system can be the target for the modulation of ferroptosis. For example, the activity of SCL7A11 can be inhibited by AMPK-mediated BECN1 phosphorylation, hence promoting ferroptosis and meanwhile inducing the progress of autophagy (24, 35). BECN1 mRNA can be stabilized by m6A modification. Furthermore, the role of YTHDF1 has been identified as a key m6A reader protein for BECN1 mRNA stability and therefore proved to activate autophagy *via* recognizing the m6A binding site within BECN1 coding regions and regulate ferroptosis (36). Besides, a CD44 variant (CD44v) can promote GSH synthesis by the interaction with system xc^-^ and stabilize system xc^-^ expression (37). The synthesis reaction of GSH can be catalyzed by glutamate-cysteine ligase (GCL) and glutathione synthetase (GSS) when the cystine oxidized to cysteine (Cys) or by cystathionine beta-synthase through a trans-sulfuration pathway which can be negatively regulated by *CARS1* (the important member from the aminoacyl-tRNA synthetase family) (38, 39).

Mitophagy can regulate lipid peroxidation through modulating the function of mitochondria. Most cellular ROS derives from mitochondria. ROS includes a series of byproducts of aerobic metabolism such as hydroxyl radicals (•OH), superoxide anion(O2•–), singlet oxygen (1O2) and hydrogen peroxide (H2O2). On one hand, mitophagy can selectively degrade mitochondria to clear dysfunctional organelles and decrease levels of ROS, therefore, preventing ferroptosis from the perspective of lipid peroxidation prevention. Until now, the identified cargo receptors that take part in mitophagy included CALCOCO2, OPTN, SQSTM1,TAX1BP1 and others (40). Mitochondrial ROS is important for both autophagy and ferroptosis induction, although the molecular switches which can determine the bifurcation between these two different types of RCD remain elusive (41–43). On the other hand, mitophagy can promote ferroptotic death by a mitochondrial complex I inhibitor (BAY87-2243) or heme oxygenase 1 (HMOX1). Inhibition of complex I of the mitochondrial respiratory chain can depolarize the mitochondrial membrane potential, mitophagy stimulation, ROS increase and cellular ferroptotic death (44). HMOX1 can mediate redox regulation of ferroptosis with enhanced endoplasmic reticulum (ER) stress and mitophagy (**Figure 1E**) (45).

The role of autophagy in modulating the absorption, utilization and export of iron is still under exploration. For example, the autophagic degradation of transferrin receptor (TFRC) (which can combine with transferrin and release iron [Fe^2+^] from transferrin into the cytoplasm through solute carrier family 11 member 2 [SLC11A2]) has been proven to be impaired due to WDR45 mutation and promote ferroptosis (**Figure 1G**) (46).

### 2.2 The role of non-selective autophagy in ferroptosis

#### 2.2.1 The role of non-selective autophagy in regulating the level of iron

**Figure 2:**
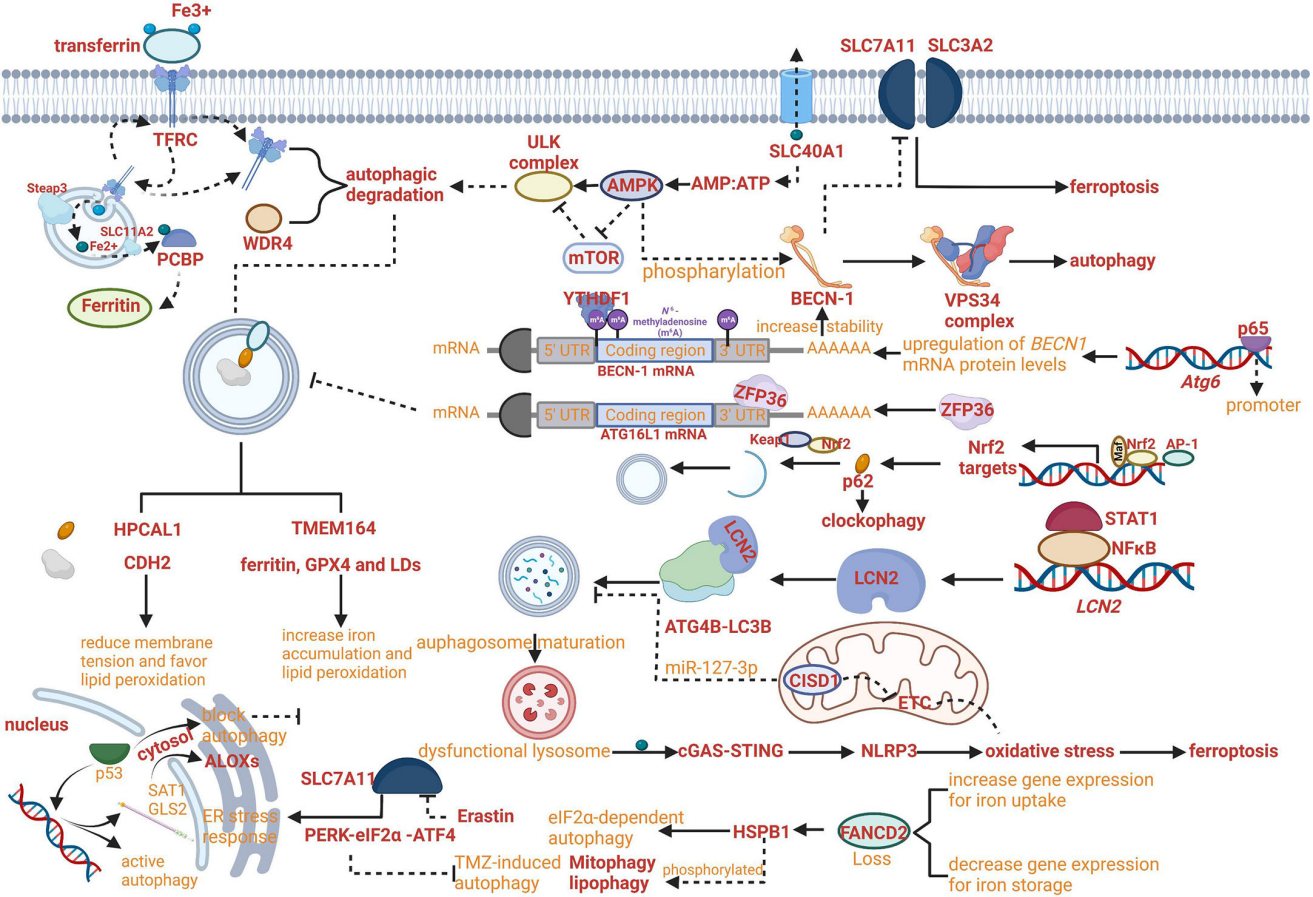
Crosslinks between ferroptosis and autophagy. **(A)** The regulatory network of iron export affecting the progress of ferroptosis and the AMP : ATP ratio affecting the progress of autophagy has been crosslinked. **(B)** NF-κB family member p65/RelA can increase autophagy coupled with upregulating levels of *BECN1* mRNA and protein. **(C)** RNA-binding protein ZFP36/TTP can inhibit autophagy activation by destabilizing autophagy related 16 like 1 (ATG16L1) mRNA. **(D)** The Keap1-Nrf2 system has been proven to involve in the phosphorylation of p62 on the cargo. **(E)** Increased lipocalin 2 (LCN2) can reduce autophagy flux by regulating ATG4B activity and LC3-II lipidation and activate inflammasome-ferroptosis processes. **(F)** HSPB1 disrupts STAT3/PKR complex, facilitates PKR-dependent eIF2α phosphorylation and activates eIF2α-dependent autophagy. **(G)** Erastin-induced ferroptosis can promote the activation of the endoplasmic reticulum (ER) stress response that is regulated by PERK-eIF2α (eukaryotic initiation factor 2α)-ATF4 (activating transcription factor 4) pathway which can inhibit TMZ-induced autophagy. **(H)** HPCAL1 as an autophagy receptor for the selective degradation of cadherin 2 (CDH2) can increase susceptibility to ferroptosis.

Iron export as the connection between non-selective autophagy and ferroptosis provided various molecular targets. Fe^2+^ can be exported as ferritin through exosomes, or by SLC40A1 in the cell membrane. Furthermore, the overexpression of SLC40A1 has been proven to activate the autophagy flux *via* AMPK/mTOR/ULK1 and AMPK/ULK1 signaling pathways to meet the energy requirements of cell and decrease the ratio of AMP : ATP (47). The regulatory network of iron export affecting the progress of ferroptosis and the AMP : ATP ratio affecting the progress of autophagy has been crosslinked and the key molecule SLC40A1 may become a critical target for regulating both ferroptosis and autophagy (**Figure 2A**).

#### 2.2.2 The role of non-selective autophagy in modulating lipid peroxidation

The role of autophagy in modulating lipid peroxidation needs to be further explored from the perspective of regulating the biosynthesis of PUFAs (the substrate of the reaction catalyzed by acyl-CoA synthetase long-chain family member4 [ACSL4] and lysophospholipid acyltransferase-3 [LPCAT3]). AMPK regulation of ferroptosis has been proven to play a critical role in PUFAs biosynthesis and phosphorylation of acyl-CoA carboxylase with the analysis of functional and lipidomic (43). Both the progress of iron export and lipid biosynthesis have a tight connection with AMPK which is also the principal enzyme to stimulate the ULK complex. Doxorubicin (Dox) cardiotoxicity- induced ferroptosis can be alleviated by epigallocatechin-3-gallate due to the upregulation and activation of AMP-activated protein kinase α2 (48). The underlying mechanism can link the increased energy supply to the modulation of ferroptosis. PUFA-PLs catalyzed by ASCL4 and LPCAT3 can be further oxidized by multiple oxygenases, for example, CYP/CYP450, PTGS/COX (prostaglandin-endoperoxide synthase), and ALOXs to produce the hydroperoxides AA-PE-OOH or AdA-PE-OOH which result in the immediate cause of lipid peroxidation (**Figure 1G**). ALOXs are nonheme iron dioxygenases and have 6 subtypes in humans, namely ALOX12 (arachidonate 12- lipoxygenase, 12S type), ALOX12B (arachidonate 12- lipoxygenase, 12 R type), ALOX15, ALOX15B (arachidonate 15-lipoxygenase type B), ALOX5 (arachidonate 5-lipoxygenase)and ALOXE3 (arachidonate lipoxygenase 3) (49).

Lipid peroxidation can also be mediated in a non-enzymatic manner through the Fenton reaction in which Fe2^+^ reacts with H2O2 to generate Fe3^+^, HO·, and OH-. The membrane lipids can be oxidized by these free radical ions (**Figure 1G**). Both the generator of ROS through the Fenton reaction and several heme or nonheme iron-containing enzymes have a close relationship with iron accumulation. Therefore, the role of autophagy in modulating the absorption, utilization and export of iron may be recognized from the perspective of affecting the process of lipid peroxidation.

### 2.3 The novel and comprehensive role of autophagy in ferroptosis

The growing evidence proved that the role of autophagy in ferroptosis regulation was not limited exclusively to aforementioned mechanism. For example, the novel regulation mechanism can be based on reducing membrane tension facilitated by hippocalcin like 1 (HPCAL1). The autophagy receptor for the selective degradation of cadherin 2 (CDH2) can reduce membrane tension and favor lipid peroxidation (Fenton reaction) to increase susceptibility to ferroptosis (**Figure 2H**) (50). In addition, increased lipocalin 2 (LCN2) can reduce autophagy flux by regulating ATG4B activity and LC3-II lipidation and activate inflammasome-ferroptosis processes (**Figure 2E**) (51).

With the development of research, the increasing roles of autophagy have been reported to be comprehensive with multiple crosslinked mechanisms for regulating ferroptosis. For example, TMEM164- mediated autophagy can increase iron accumulation and lipid peroxidation by degrading ferritin, GPX4 and LDs (52). Insufficient cellular autophagy can turn off antioxidant defense mediated by nuclear factor NF-E2-related factor (Nrf2) while initiating Nrf2-induced iron accumulation and lipid peroxidation, resulting in the advancement of ferroptosis. The Keap1-Nrf2 system has been proven to involve in the phosphorylation of p62 on the cargo, which can regulate clockophagy to affect the process of ferroptosis (**Figure 2D**) (53). However, the relevant signaling pathways need to be further explored (54).

Meanwhile, the regulatory network of autophagy-dependent ferroptosis was enriched. For example, RNA-binding protein ZFP36/TTP can inhibit autophagy activation by destabilizing autophagy related 16 like 1 (ATG16L1) mRNA *via* binding to the AU-rich elements (AREs) within the 3′-untranslated region. The downregulation of ZFP36 can activate ferritinophagy and induce ferroptosis by regulating the signaling pathway of autophagy (**Figure 2C**) (55).

## 3 The role of ferroptosis in autophagy

Autophagy is a dynamic process relying on the maturation and formation of specific membrane structures including phagophores, APs, and ALs, which can be generated from the plasma membrane, Golgi complex, recycling endosomes and the ER mitochondria-ER-associated membrane (56, 57). Mechanistically, ATG proteins play an indispensable role in the regulation of autophagy concerning initiation, progression and maintenance. Genetic screens in yeast have identified over 40 ATG genes regulating the expression of ATG proteins which can interact with other factors by multiple posttranslational modifications (58). The phagophore and AP formation can be governed by the joint influence of both ATG proteins and other factors. It has been proved that synaptosome-associated protein29 (SNAP29), the homotypic fusion and vacuole protein sorting (HOPS) complex, vesicle-associated membrane protein 8(VAMP8), regulatory lipids, certain cytoskeleton motor proteins, syntaxin 17 (STX17) are involved in the formation of AL (59).

Although the direct evidence supporting the critical role of ferroptosis in autophagy is limited, the hypothesis that ferroptosis can regulate autophagy has been confirmed gradually based on the molecular connection between ferroptosis and autophagy. Indeed, erastin-induced ferroptosis can promote the activation of the endoplasmic reticulum (ER) stress response that is regulated by PERK-eIF2α (eukaryotic initiation factor 2α)-ATF4 (activating transcription factor 4) pathway which can inhibit TMZ-induced autophagy (**Figure 2G**) (60, 61). Besides, it has been reported that the process of protective autophagy can be induced by iron deprivation with antitumor drugs, which can also be reversed by ferric ammonium citrate (FAC) through iron supplementation (62).

The regulation of autophagy may have a tight connection with ferroptosis in the context of inducing different immune and inflammatory reactions, increasing lipid peroxidation or ROS products (which may impair the function of lysosome to suppress autophagy or induce the damage of mitochondria to initiate the subsequent onset of autophagy), and affecting the common signal transduction pathways.

For example, the lipid peroxidation product 4HNE as a pro-inflammatory mediator can activate the nuclear factor-κB (NF-κB) pathway which is a crucial regulator in the context of monocyte-to-macrophage differentiation through autophagy (63, 64). In the promoter of the human *BECN1*autophagic gene (*Atg6*), a conserved NF-κB binding site has been found. Therefore, the NF-κB family member p65/RelA can increase autophagy coupled with upregulating levels of *BECN1* mRNA and protein in different cellular systems (**Figure 2B**) (65). Meanwhile, the NF-κB pathway can be activated by a pattern-recognition receptor, advanced glycosylation end-product-specific receptor(AGER/RAGE), in peripheral macrophages by HMGB1 (a typical DAMP participating in multiple types of cell death released by ferroptotic cells) (66, 67). DAMPs such as HMGB1 result in chemotherapy resistance coupled with the upregulation of autophagy (68).

The common signal molecules or pathways also play a critical role in the crosslinks between ferroptosis and autophagy. For example, in the cytosol, p53 can block autophagy in a transcription-independent manner, whereas in the nucleus, p53 can activate autophagy in a transcription-dependent way (69). Meanwhile, nuclear p53 can accelerate the expression of glutaminase 2 (GLS2) and spermidine/spermine N1-acetyltransferase 1 (SAT1) which can induce lipid peroxidation through ALOXs (70). Furthermore, the activity and expression of SLC7A11 can be negatively regulated by p53 resulting in ferroptosis, whereas p53 has been proved to antagonize ferroptosis by the formation of the dipeptidyl-peptidase-4 (DPP4)-p53 complex (71, 72). When the ability of DPP4 to form NADPH oxidase 1 (NOX1) complexes with NOX1 in the nucleus is blocked, ROS production can be reduced and ferroptosis can be inhibited (73). p53-mediated ferroptosis can be upregulated by acetylation of p53 (74). However, the induction of p53 deacetylation, due to either the activation of the deacetylase Sirtuin 1 (Sirt1) or the mutation of the acetylated lysine site in p52 can promote autophagy (75).

Another critical crosslinked signaling pathway was STAT3/Nrf2/GPX4. On one hand, impairing STAT3/Nrf2/GPX4 signaling pathway can reactivate ferroptosis, which can be used to attenuate drug resistance (76). On the other hand, cytoplasmic STAT3 can suppress autophagy through binding to protein kinase B (PKB) (the inhibition of PKB/Akt can be induced by the activation of mTORC1 and result in the inhibition of autophagy), in turn, promote mitochondrial localization of STAT3 and its phosphorylation induced by IL-6 (77, 78).

Some crucial crosslinked molecules have been explored to find the connection between ferroptosis and autophagy. The mitochondrial protein, CDGSH iron sulfur domain 1 (CISD1, also called mitoNEET) can mediate the crosstalk between oxidative stress and mitochondrial iron uptake in the outer membrane of mitochondria. The expression of CISD1 has a tight connection with both autophagy and ferroptosis. The overexpression of CISD1 can effectively inhibit autophagic cell death, which can be modulated by a specific regulator miR-127-3p (79–81). In addition to regulating autophagy, overexpression of CISD1 can also limit ferroptosis by a guard against lipid peroxidation induced by ROS from mitochondria (82).

Besides, heat shock protein family B (small) member 1 (HSPB1, also called HSP27 in humans or HSP25 in mice) was confirmed to be central in regulation of autophagy and ferroptosis, which is a molecular chaperone with a function in counteracting protein misfolding and aggregation. The mutations in *Hspb1/HSP25*, both targeting its catalytic alpha-crystallin domain and the C-terminus, can downregulate autophagy levels (83). In detail, HSPB1 disrupts STAT3/PKR complex, facilitates PKR-dependent eIF2α phosphorylation and activates eIF2α-dependent autophagy (**Figure 2F**) (84).The phosphorylated HSPB1 involves in mitophagy and lipophagy (85, 86). It has also been found that phosphorylated HSPB1 induced by erastin can block cytoskeleton-mediated iron uptake and subsequent lipid peroxidation under ferroptosis (**Figure 2F**) (87). Previous studies have confirmed that the expression of HSBP1 can be upregulated with the loss of FANCD2 which is the central protein of the Fanconi anemia (FA) pathway (88). In addition, loss of FANCD2 is also closely related to increased gene expression for iron uptake (such as transferrin and transferrin receptor) and decreased gene expression for iron storage (such as FTH) and iron export (such as hepcidin antimicrobial peptide) in ferroptosis (89). Meanwhile, FANCD2-deficient cells have been proven to behave hypersensitive to oxidative stress and impaired autophagy leading to DNA crosses links (90).

Another principal crosslinked target called NEDD4 (neural precursor cell expressed developmentally down-regulated protein 4) is a member of the HECT E3 ubiquitin ligases, which is closely related to the mTOR signaling pathway and promotes autophagy (91). Meanwhile, NEDD4 can also regulate oxidative damage and iron metabolism by the degradation of voltage-dependent anion channels (VDAC) (which can mediate the transport of ions and metabolites in eukaryotic cells across the outer membrane of mitochondria) and lactotransferrin (LTF) (that can specifically bind and transport iron) (92, 93).

## 4 The crosslinks between ferroptosis and autophagy in asthma

Both autophagy and ferroptosis are involved in various diseases, and crosslinks between autophagy and ferroptosis in various diseases have been emerging. Autophagy and ferroptosis can be initiated as a defense against varieties of intra- and extra-cellular stress stimuli, which can be achieved in large part through a synergistic immune response. The immune response can lead to alterations of multiple signal molecules which may induce a new round of ferroptosis or autophagy of various cells. The crosslinked intra- and extra- signal molecules or pathways affect the function of leukocytic or non-leukocytic cells and influence the progress of various diseases. The deepened cognition of the role of crosslinks between ferroptosis and autophagy in diseases may provide new targets for therapy, novel signal pathways associated with the pathogenesis of the disease, innovative paradigms to recognize and regulate the immune and inflammatory reactions and in general therapeutic benefits for patients.

Although the links between asthma and ferroptosis or autophagy have been confirmed, the role of crosslinks between these two forms of RCD remains unclear. In the latter section, we demonstrate recent advances in the evolving comprehension of the interface between autophagy, ferroptosis and asthma from the perspective of functional cells involved in the pathogenesis of asthma. We discuss how the crosslinked signal pathways in immune or non-immune cells affect the pathogenesis of asthma, how these two forms of RCD reciprocally induce the occurrence of each other through immune and inflammatory signals, how emerging concepts about the crosslinks between ferroptosis and autphagy reshape our understanding of immunity and asthma.

IL-13 can increase LC3II expression and then induce autophagy. Meanwhile, IL-13 can induce ferroptosis. IL-33 can activate autophagy through the inhibition of mTORC1. Autophagy can decrease the level of p62 and increase secretion of IL-18.

### 4.1 The crosslinks and HAECs

**Figure 3:**
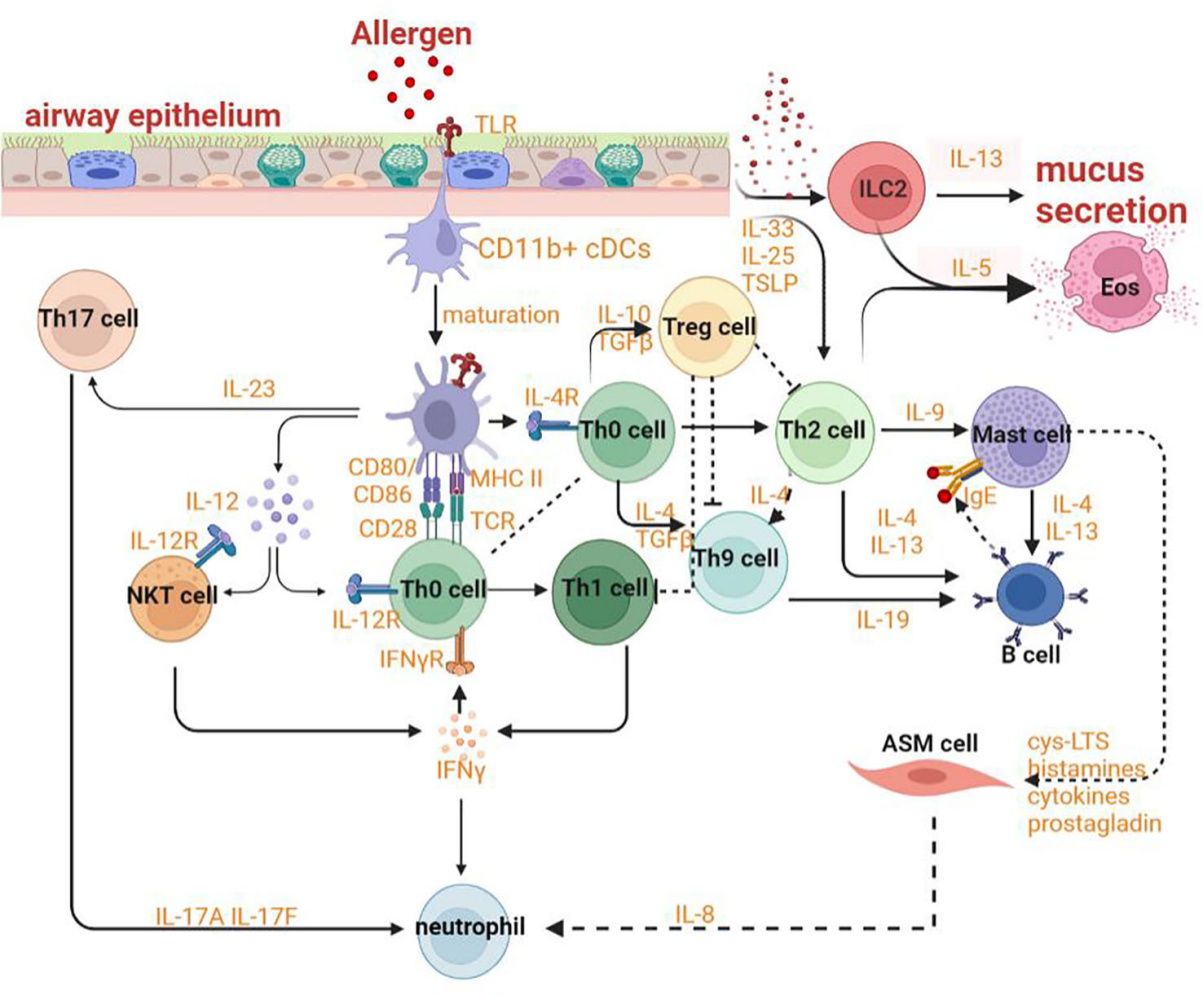
Mechanism of asthma.

HAECs can promote the regeneration of tissues and protect the body from stimuli, allergens and pathogens by releasing inflammatory response mediators and cytokines such as thymic stromal lymphopoietin (TSLP) and chemokine (C-X-C motif) ligand (CXCL)-8, CXCL1, IL-25, IL-33 and activating innate and adaptive immune systems. The various allergens and proteases can induce the expression of the epithelial cytokines and then promote Th2 immunity by activating conventional dendritic cells (cDCs) and by activating innate lymphoid type-2 (ILC2) cells and basophils that could efficiently polarize IL-4 and/or IL-13 for promoting Th2 immunity and decreasing tolerance to inhaled allergens (94–98). In ongoing asthma, HAECS continue to fuel inflammation in the airway by activating incoming monocytes to adopt an immunogenic phenotype and by generating cytokines and chemokines to activate neutrophils, eosinophils, and other cells of the innate immune system (**Figure 3**) (99). Epithelial cells also substantially contribute to airway remodeling by releasing repair cytokines along with the repeated cycles of injury and repair (100).

**Figure 4:**
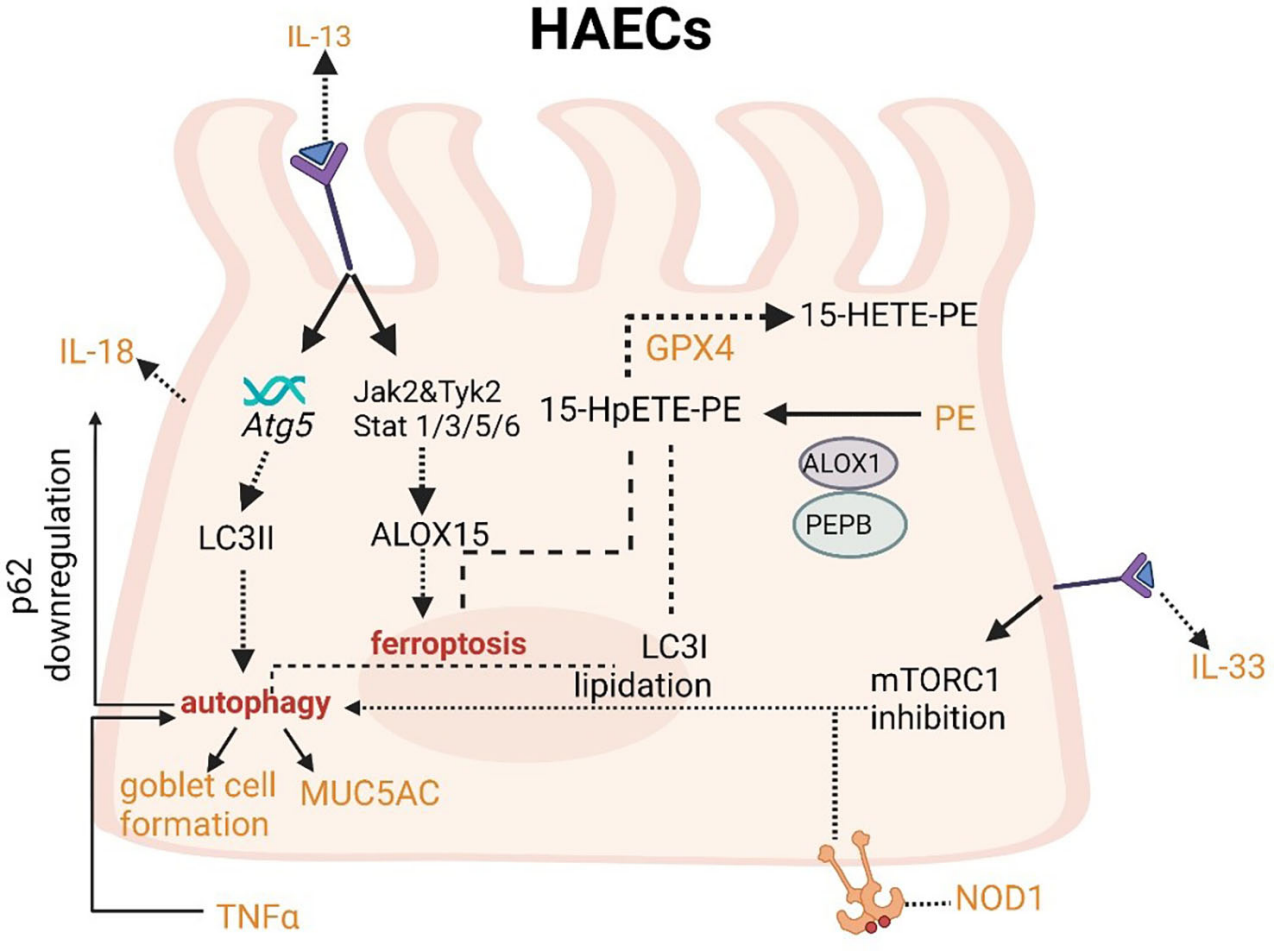
Crosslinks between ferroptosis and autophagy in HAECs.

The upregulation of IL-13 can not only increase autophagic flux (that can be prevented by *Atg5* knockdown) and expression of LC3-II in order to induce autophagy and then stimulate goblet cell formation and MUC5AC secretion from HAECs (**Figure 4**), but also promote the occurrence of ferroptosis by upregulating the expression of ALOXs such as ALOX5 and ALOX15 (**Figure 4**) (101, 102). When blocking autophagy in HAECs, the generation of ROS can be inhibited through the activation of the NADPH oxidase DUOX1 (103). The suppression of ROS production can also negatively regulate the process of ferroptosis. NOD-like receptor (NLRs) signaling pathway mediated activation of HAECs interacting with eosinophils, which may trigger allergic asthma (104). Nucleotide-binding oligomerization domain-containing protein 1 (NOD1) and NOD2 that can initiate NF-κB-dependent and mitogen-activated protein kinase (MAPK)-dependent gene transcription are crucial for innate immune responses (105). NOD1 agonists have been proven to induce autophagy in HAECs and the activation of NOD1 can increase the levels of GPX4 and other iron and ferroptosis regulatory proteins in macrophages (106, 107). Cellular ferroptotic death has been proven to be more likely to trigger inflammatory signal molecules than apoptotic death by the less effective macrophages (108). TNFα as a critical pro-inflammatory cytokine produced by macrophages in the airway lumen and bronchial mucosa can promote the initiation of the HAECs autophagy (109). Autophagy in HAECs can decrease the level of p62 the autophagy receptor for clockophagy and downregulate the level of ferroptosis, which has a tight connection with increased secretion of IL-18 (**Figure 4**) (110). Inflammatory response mediators and cytokines released by HAECs can activate the innate and adaptive immune systems such as chemokine (C-X-C motif) ligand (CXCL)-8, CXCL1, thymic stromal lymphopoietin (TSLP), IL-33, and IL-25. Thereof, IL-33 can activate autophagy through the inhibition of mTORC1 and be inhibited by *LC3B* knockdown (**Figure 4**) (111).

In mature DC, ATG5 and MAP1LC3B involve in the regulation of TLR stimulation. ATG7 involves the regulation of the STING signal pathway which can promote autophagy through the lipidation of MAP1LC3. The signal pathway of TLR4 can induce NF-κB–dependent proinflammatory cytokines. When TLR4 binds to NOX4 or its isoenzymes, ferroptosis would be induced. HMGB1 can promote autophagy by releasing the basic autophagic gene Beclin 1. CMA contributed to the antigen-presenting process through overexpression of LAMP2A.

In T cells, Beclin1 has been shown to negatively modulate the generation of Th2 cytokines IL-5 and IL-13 and upregulate the production of IL-17 and IFN-γ by co-culturing CD4^+^ T cells. After being activated by TCR/CD28 co-stimulation, Treg cells can induce the expression of GPX4. CD8^+^ T cells can induce ferroptosis of surrounding macrophages and other activated immune cells to accelerate inflammation through IFN-γ which can down-regulate the expression of SLC7A11 and SLC3A2.

### 4.2 The crosslinks and antigen-presenting cells in the innate immune system

The interaction between epithelial cells and DCs plays an indispensable role in sensitization to allergens and the advancement of asthma. Both HAECs and lung cDCs can express pattern-recognition receptors and can be directly activated by allergens (112). CD11b^+^ cDCs can maturate and migrate depending on the transcription factor IRF4 with the effect of danger signals and ‘instructive’ cytokines produced by HAECs (**Figure 3**) (113). After stimulation by ILC2 cells secreting IL-13, these cells then would induce Th2 and Th17 responses in draining mediastinal lymph nodes (114). With the help of basophils, DCs can sustain Th2 responses in the lymph nodes. When the lung is exposed to allergens repeatedly, CD11c^hi^ DCs will actively participate in the TH2 effector phase of asthma (115). After being stimulated by allergens, effector Th2 cells could be recruited by CCL22 and CCL17 produced by macrophages and/or CD11c^hi^ monocytic DCs with poor migration capability (115). Poorly migratory CD11c^hi^ monocytic DCs have some similar features as macrophages, for example, the ability to express FcϵRI and CD64 (115).

**Figure 5:**
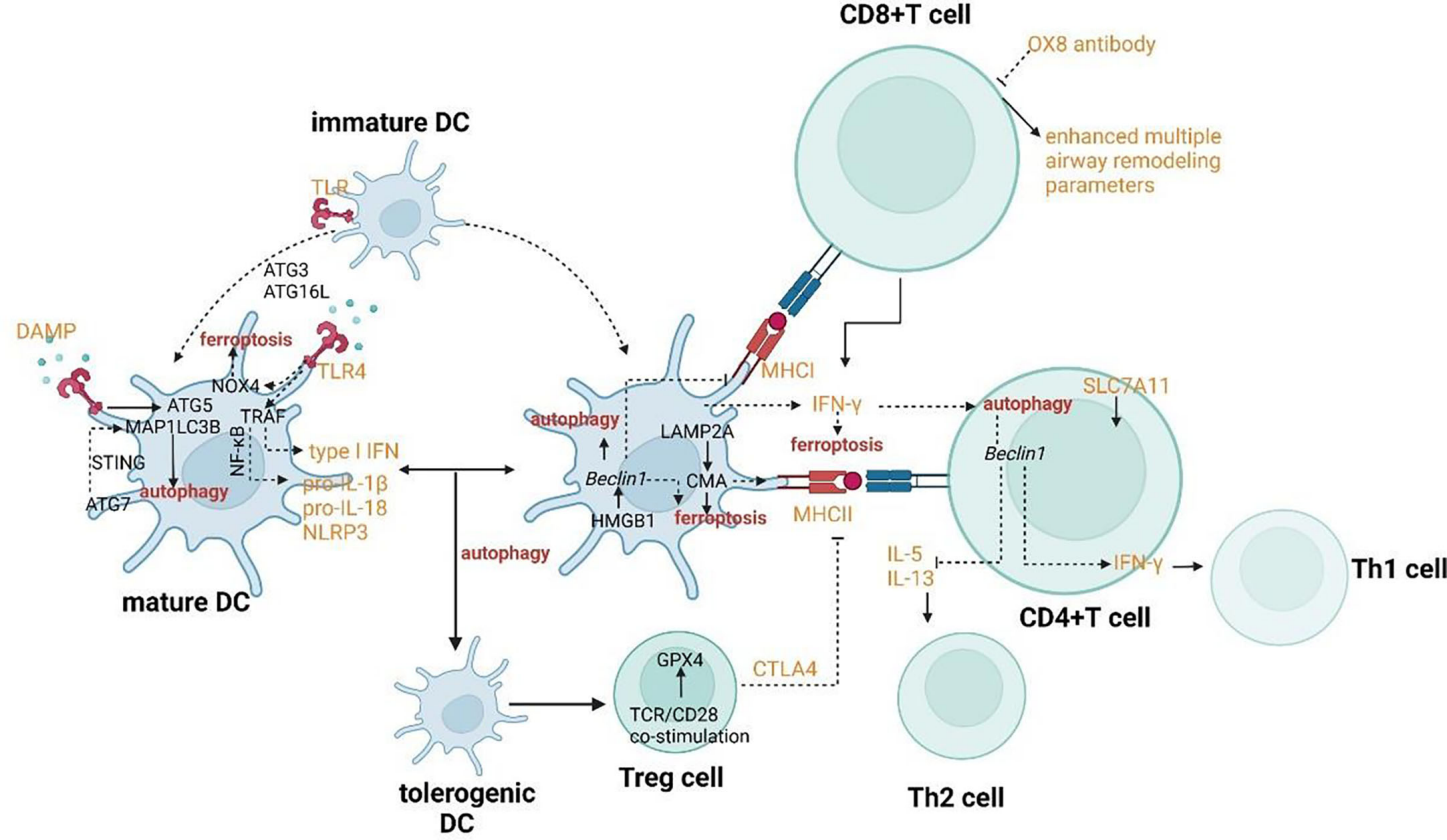
Crosslinks between ferroptosis and autophagy in dendritic cells and T cells.

The crosslinks between ferroptosis and autophagy can affect DCs in the context of functional maturation, migration, antigen presentation, and MHC-II presentation of extracellular (phagocytosed) antigens. DCs endure two opposite functional and phenotypic states of maturation. The maturation from a tolerogenic state to the activated one occurs with the help of pathogen-associated molecular patterns (PAMPs), the most characterized type of TLRs. ATG5 and MAP1LC3B regulate TLR stimulation to promote the maturation of DCs (**Figure 5**) (116). Both two autophagy proteins also involve in TLR4-mediated responses in DCs. The signal pathway of TLR4 can promote the innate immune responses by inducing NF-κB–dependent proinflammatory cytokines after activated by adaptor MYD88 or by accelerating the generation of type I IFN after activated by the adaptor TCR adaptor molecule 1 (TICAM1/TRIF). Furthermore, when TLR4 binds to NOX4 or its isoenzymes, TICAM1 would be activated and ferroptosis would be induced (117). On one hand, autophagy can regulate TLR signaling transduction by acting upstream. On the other hand, autophagy can be modulated by the activation of TLR. For example, the initiation of TLR4 has recently been proven to inhibit autophagy due to the activation of mTORC1 (118). However, the combinatorial initiation of NOD2 and TLR4 has been proven to promote autophagy, indicating that the impact of NOD2 on the regulation of autophagy is predominant over TLR4 initiation (**Figure 5**) (119). The maturation endows DCs with the migratory capability to activate naïve T cells and promote effector T cell responses in secondary lymphoid tissues. Migration of DCs can also be regulated through autophagy affecting the modulation of their cytoskeleton (120). ATG7 and ATG16L can regulate the recruitment and migration of CDs to promote their communication with lymphocytes and to coordinate the adaptive immune responses. ATG7 also involves IFN-α secretion by DCs. It has been confirmed that the induction of IFN-αwas facilitated by the cGAS-STING signal pathway (121–123). Hence, it can be concluded that ATG7 involves the regulation of the STING signal pathway which can promote autophagy through the lipidation of MAP1LC3 (**Figure 5**). Furthermore, the release of 8-OHG from ferroptotic cells can co-activate the STING1-dependent inflammatory pathway (124).

The typical function of DCs as antigen-presenting cells is to internalize antigens and present antigen-derived peptides to T cells. The exogenous and endogenous can be presented by DCs to CD4^+^ T and CD8^+^ T cells by MHC class II and class I, respectively. MHC-I can present the extracellular proteins by a process called cross-presentation. CMA has been proved to contribute to the antigen-presenting process by mediating the translocation of substrates across the lysosomal/late endosomal membrane during CMA through overexpression of LAMP2A (**Figure 5**) (125). Therefore, the process of antigen presentation involved with CMA may also result in the ferroptosis of DCs. When DCs exposed to the ferroptotic cells, the antigen processing and presentation by DCs may reduce (126). In process of MHC class II-restricted antigen presentation, the cytosol galectin 8 can directly recruit the macroautophagic machinery for lysosome degradation of the damaged endosome and the ubiquitinated endosome cargo can recruit the LC3 anchor proteins such as p62 for targeting to APs (127–129). The signal pathway of autophagy-dependent ferroptosis is also involved in extracellular antigen processing for MHC-I presentation. Some specific types of allergen can be surrounded by IFN-γ collaboration with autophagy proteins such as ATG3, ATG5, ATG7 and ATG16L1 so that the vacuolar membrane can be broken and the allergen can be exposed to the cytosol and degraded successively, possibly by autophagy (130). The exposure results in p62 association with the allergen, which can promote CD8^+^T-cell responses (131). The lower secretion of IFN-γin DCs may result from haploinsufficiency of an essential protein, Beclin-1 and result in the downregulation of MHC-I presentation and the suppression of system xc^-^ which can induce ferroptosis (132, 133). In addition to IFN-γ, HGMB1 also plays a crucial role in the coordination of ferroptosis and autophagy in the innate immune system. When mature DCs secrete this leaderless cytokine, T-cell and B-cell responses can be activated (134). Meanwhile, the prototypical DAMP involves the stimulation of inflammatory response in peripheral macrophages by activating AGER/RAGE (67). HMGB1 can also promote autophagy by releasing the basic autophagic gene Beclin 1 from the BCL-2 complex (**Figure 5**) (86).

**Figure 6:**
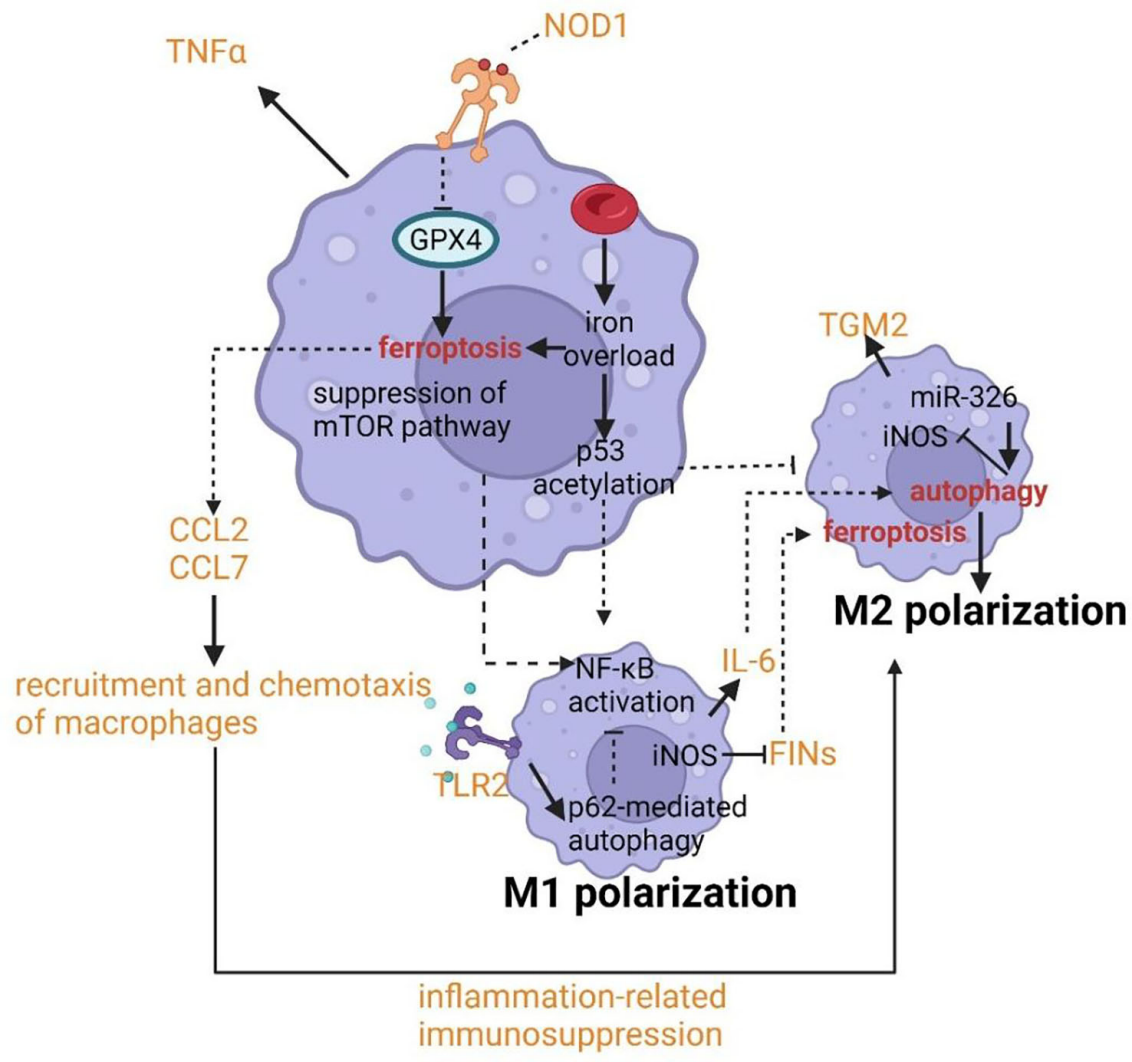
Crosslinks between ferroptosis and autophagy in macrophages.

The polarization of macrophages has been shown to contribute to the pathogenesis of asthma. Typically, macrophages can be polarized into the M1 phenotype with increased cellular immunity and pro-inflammatory cytokines production by IFN-γ or LPS and M2 phenotype with increased anti-inflammatory responses to promote tissue repair and humoral immunity (135, 136). Nowadays, the bimodal subdivision has been abandoned in favor of a model of a spectrum of polarization changes in macrophages that better illustrates the great variety in macrophage responses to stimuli (137). The alteration of cellular processes such as efferocytosis, phagocytosis, and (anti-) inflammatory cytokine generation results in the pathology of asthma. The crosslinks between ferroptosis and autophagy provide us with novel targets and new therapeutic strategies by regulating the function and polarization of macrophages. p53 acetylation and ROS production due to iron overload can lead to M1 polarization with the upregulating expression of M1 markers including IL-1β, IL-6, and TNF-α and decreasing levels of M2 markers such as TGM2 (**Figure 6**) (138, 139). M1 polarization can also be stimulated by the suppression of the mTOR pathway which is the master controller of autophagy (140). NF-κB can be activated after M1 polarization, in fact, the activation of NF-κB is able to drive macrophages to either M1 or M2 polarization (141, 142). NF-κB p65 cytosolic ubiquitination induced by TLR2 signal can result in its degradation by p62-mediated autophagy. The repression of autophagy can rescue the activity of NF-κB and drive macrophages to M2 phenotype (143, 144). Similarly, IL-6 and CCL2 can trigger M2 phenotype by inducing autophagy in macrophages (**Figure 6**) (145). The chemotaxis and recruitment of macrophages can be assisted by CCL2 and CCL7 regulated by ferroptosis inducing expression of inflammation-related genes (146–148). In addition, along with the occurrence of ferroptotic cellular death, inflammation-related immunosuppression can be formed through macrophage polarization (149). Inducible nitric oxide synthase (iNOS) as the critical marker of macrophage M1/M2 polarization has been uncovered to have a potential relationship with ferroptosis and autophagy. The overexpression of miR-326 can promote autophagy along with the downregulation of iNOS expression (149). And the higher activity and enrichment of iNOS in M1 phagocytes compared to M2 phagocytes confers higher resistance to ferroptosis induced by reagents (150). An important feature of asthma is the altered colonization of microbes resulting from the defective phagocytosis of monocytes and macrophages. Hence both limiting immune activity and defective phagocytosis will promote the development of asthma due to cellular ferroptotic death or defects of LC3-associated phagocytosis (LAP). Unluckily, ferroptosis of macrophages has been proven to be triggered by iron overload when scavenging aged erythrocytes (151). LAP can promote antigen presentation to T cells by MHC-II and help to clear pathogens *via* engulfment and phagosome acidification (152–154). Therefore, the deepened cognition of signal pathways and molecules associated with LAP such as the TLR9 pathway, TRAF3 and IRF7 may provide novel targets for asthmatic therapy.

IL-6 can trigger M2 phenotype by inducing autophagy in macrophages. The chemotaxis and recruitment of macrophages can be assisted by CCL2 and CCL7 regulated by ferroptosis inducing expression of inflammation-related genes. p53 acetylation can lead to M1 polarization with the upregulating expression of M1 markers including IL-1β, IL-6, and TNF-α and decreasing levels of M2 markers such as TGM2.

### 4.3 The crosslinks and the adaptive immune system

With the deepened research on asthma, the paradigm of cognition on asthma has gradually transformed from a single disease into a syndrome (101, 155). The endotypes which are defined as distinct pathophysiology due to different asthma phenotypes can differ in terms of genetic susceptibility, age of onset, clinical presentation, environmental risk factors, prognosis and response to standard and new therapies (156, 157). The underlying immunological basis of multiple asthma endotypes has a close relationship with the adaptive immune system, especially with T cells. When activated by the innate immune system, CD4^+^T cells can differentiate into multiple functional subsets of helper T cells such as Th2 cells, Th17 cells, Th1 cells, and Treg cells. Both Th2 cells and ILC2 cells contribute to eosinophilic inflammation by upregulating the expression of GATA-3 which can promote the production of Th2 cytokines, upregulate the expression of chemokine receptors such as CCR4, CCR8 and CRTH2, and increase the production of IL-5 to modulate the development of eosinophils, IL-13 leading to goblet cell metaplasia and bronchial hyperreactivity, IL-4 affecting the mature and activation of Th9 cells which can promote the IgE synthesis by B cells. Both Th1 and Th17 cells can result in neutrophilic inflammation by respectively secreting IFN-γ or TNFαand IL-17A or IL-17F Treg cells, a subset of CD4^+^ T cells, also originate from Th0 cells and can express transcription factor forkhead box P3 (Foxp3) and IL-2 receptor (CD25) to suppress allergic responses (**Figure 3**). The fate of follicular helper T cells (TFH) can be adopted by Th cells producing IL-21 so that IgE can be induced by B lymphocytes. DCs can sustain Th2 responses with the help of basophils in lymph nodes. The crosslinks regulation of ferroptosis and autophagy in adaptive immunity including in antigen presentation, mutation and activation of effector cells, and immune signaling regulation may provide us with new modulatory strategies and novel targets to effectively alleviate asthmatic inflammation and symptoms.

The role of ferroptosis and autophagy in antigen presentation of CD4^+^ T cells can be recognized from MHC class II presentation perspectives, DC-mediated T cell activation, and some key signaling molecule production. MHC class II antigens can derive from both extracellular and intracellular sources. Thereof, autophagy plays an essential role in delivering materials into lysosomes to promote the generation of intracellular sources for MHC class II antigens. In addition, autophagy involves the antigens’ capture after their evasion from phagosomes and delivers them into lysosomes to promote CD4^+^ T cells (158). The formation of AP-like structures has been reported to emanate from MHC class II compartments (MIICs) in DCs, which contain the markers of AP Atg16L1 and LC3 as well as the principal molecular machinery involved in antigen-processing (159). The phagocytosis of macrophages can be regulated by the iron accumulation, generation of lipid peroxidation and the release of ROS, which means that ferroptosis can modulate the MHC class II antigens production through alteration of the derivation of MHC class II antigens. The production of MHC class II can be downregulated by cytotoxic T lymphocyte antigen 4 (CTLA-4) and other inhibitory molecules secreted by Treg cells. Meanwhile, Treg cells can induce CD4^+^ T cells to produce anti-inflammatory cytokines such as TGF-β and IL-10 (160). The upregulated amount of CD4^+^CD25^+^Treg cells has been reported in asthmatic patients with corticosteroid therapy (161). Therefore, if the crosslinks between autophagy and ferroptosis can effectively regulate the activity and amount of the inflammatory T cells and anti-inflammatory T cells, the prognosis of asthma may be improved drastically. Much research has been conducted to find the distinguished receptors which may be the potential targets for asthmatic therapy if the effect is poles apart after activated by common signal molecules regulating ferroptosis or/and autophagy on or in different subsets of T cells. In human naive CD4^+^ T cells, SLC7A11 has been proven to be deficient. However, when CD4^+^ T cells are activated, the ferroptosis-related protein can be drastically upregulated (162, 163). Autophagy is carried out constitutively to low levels in CD4^+^ T cells and can be induced following T-cell receptor activation (164). Multiple genetic model systems have been applied to explore the role of the specific expression product in regulating the function and amount of T cells. The levels of autophagy can be upregulated in T cells with a deletion in *Atg5* ^−/−^, *Atg7* ^−/−^, *Atg3* ^−/−^ and *Vps34* ^−/−^ in the lymph nodes (164–168). In autophagy-deficient T cells, ferroptosis may also be easily induced due to the increase in mitochondrial load with enhanced levels of ROS. The reducing extracellular microenvironment is necessary for CD4^+^ T cells to activate and proliferate from the perspective of the maintenance of intracellular GSH levels. The mechanisms inspire us to modulate the heterogeneity of CD4^+^T cells or even T cells by the various distribution of sensitivity on the signal molecules of autophagy and ferroptosis in order to generally recover the balance of inflammatory and anti-inflammatory responses.

The activation of T cells has always been the focus for researchers to explore in order to explain the pathogenesis of asthma and find novel targets for treatment. The regulation of Th2 subsets has a tight connection with the function of eosinophils, while the effect on Th1 and Th17 subsets may influence the pathogenesis of neutrophilic inflammation. The activation of Treg cells can also be modulated for anti-inflammation reactions. CD4^+^ T cells can be activated by DCs through autophagy as judged by their capability to secrete IFN-γ (119). Beclin1 has been shown to negatively modulate the generation of Th2 cytokines IL-5 and IL-13 and upregulate the production of IL-17 and IFN-γ by co-culturing CD4^+^ T cells (**Figure 5**) (169). IFN-γ as a critical modulator of autophagy and activation of CD4^+^ T cells also has the crosstalk with ferroptosis. CD8^+^ T cells can induce ferroptosis of surrounding macrophages and other activated immune cells to accelerate inflammation through IFN-γ which can down-regulate the expression of SLC7A11 and SLC3A2 (**Figure 5**) (170). Although our recognition of the potential roles of CD8^+^ T cells in asthma is still limited, the positive role of CD8^+^ T cells has been demonstrated. It has been found that rats formerly sensitized and treated with OX8 antibody (which can lead to the consumption of CD8α^+^ T cells) have enhanced multiple airway remodeling parameters such as mucus production and airway smooth muscle volume, airway inflammation and epithelial cell proliferation (171–173). After being activated by TCR/CD28 co-stimulation, Treg cells can induce the expression of GPX4 (**Figure 5**) (174, 175). Deletion of GPX4 in Treg cells can result in ferroptosis and the production of IL-1β which can mediate lung neutrophilia and IL-33 expression (174, 175). The activation of Treg cells also has a tight connection with autophagy. Tolerogenic DCs can induce enhanced proliferation of CD25^+^ Foxp3^+^ Treg cells. Furthermore, the tolerance to drive the DC phenotype to tolerogenic functions is induced by autophagy (176). TCRγδ^+^ T cells have been shown to be activated resulting from the improved DC numbers and costimulatory molecule expression in Atg16L1-deficient mice (177). γδ T cells are an important subset of innate-like T cells in asthma. Another subset of this group is called natural killer T (NKT) cells. Regardless of the occurrence of Th2 cells, NKT cells can involve in allergic responses in asthma. Meanwhile, they can accelerate airway hyperresponsiveness (AHR) in the defect of adaptive immune responses, particularly in situations with viral infections or neutrophils (178). γδ T cells have a tight connection with HAECs as an immune surveillance guarder, responding to tissue damage and endogenous stress signals. Hence, a major paradox should focus on the fact that the modulation of T cells with the crosslinks between autophagy and ferroptosis may alleviate the allergic responses for asthmatic patients, while the protection against HAECs may be destroyed further because of the impaired effect of γδ T cells. The research on the signaling pathway which can regulate T cells with inflammation causing the effect but protect the function and integrality of T cells with body protective effect needs to be further explored.

B cells in asthma mainly function as antigen-presenting cells, which are crosslinked by high affinity IgE receptor FcϵRI to enhance the activity of basophils and mast cells (**Figure 3**). Antigen-specific IgE in serum can promote the pathogenesis of asthma by inducing the immediate response of basophils and mast cells. B cells are activated by IL-13 and IL-14 from Th2 cells and basophils, IL-19 from Th19 cells, IL-4 and IL-13 from mast cells. Therefore, the regulation of B cells with respect to their activation or amount can be intervened from the perspective of T cells modulation which both ferroptosis and autophagy have already been shown to involve. Besides, B cells can also promote airway inflammation and induce AHR in the absence of T cells (179). Hence, the role of crosslinks between ferroptosis and autophagy in the direct regulation of B cells is also necessary for asthmatic therapy.

**Figure 7:**
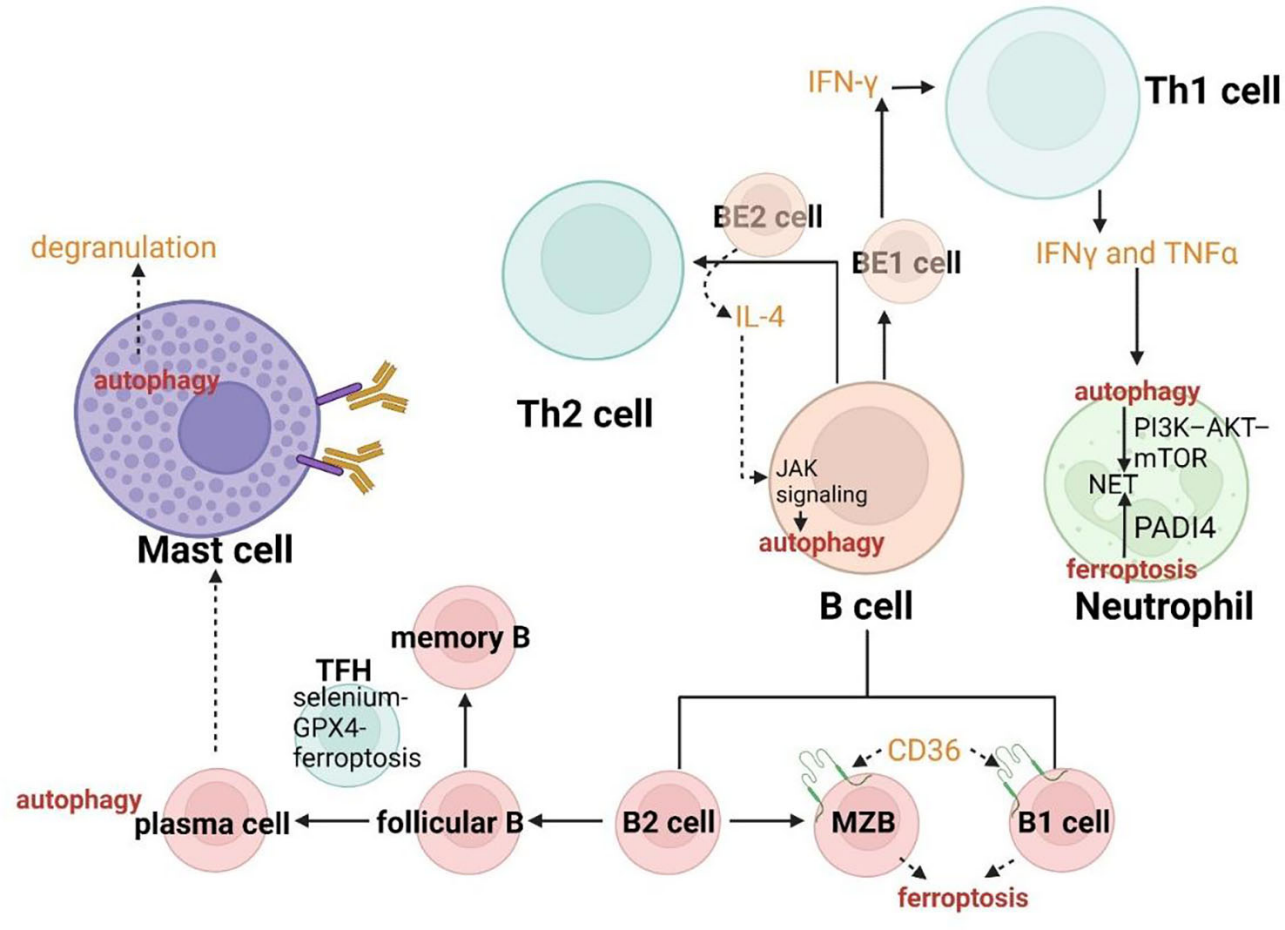
Crosslinks between ferroptosis and autophagy in B cells, mast cells and neutrophils.

The specific deletion of *Atg5* in B cells has been proved to acquire a deficient transition for these autophagy insufficient B-cell progenitors between pro- and pre-B-cell phases in the bone marrow, indicating the critical role of autophagy in B-cell development (180). In addition to the process of advancement in the center, B cells can also be severely influenced by both ferroptosis and autophagy in the periphery. B cells include two main subgroups. One of them called B1-lymphocytes (B1a and Bib lymphocytes) arising from fetal liver precursors always gather in peritoneal and pleural cavities as well as mucosal tissues. Another group called B2-lymphocytes (follicular B lymphocytes and marginal zone B (MZB) lymphocytes) derived from precursors in the bone marrow are enriched in secondary lymphoid organs. B1 lymphocytes and MZB always involve in the rapid humoral response to achieve the natural defense, while follicular B lymphocytes play an essential role in response to exogenous antigens. Compared with follicular B cells, MZB cells and B1 cells have a tighter connection with ferroptosis. The enhanced ferroptosis sensitivity and fatty acid uptake resulted from the higher expression of CD36 (the protein in charge of fatty acid transport) lead to the regulators targeting GPX4 easily inducing the activation of maintenance, progression and antibody response of B1 cells and MZB cells (**Figure 7**) (181). The role of autophagy has been confirmed to be a particular requirement in B1a cells homeostasis (182). Follicular B cells differentiate into plasma cells and memory B cells with high affinity and long life characterized by selection and mutation, through the germinal center (GC) reaction induced by TFH cells. On one hand, plasma cells require autophagy for sustainable immunoglobulin production (182). On the other hand, TFH cells have been reported to present vulnerable to ferroptosis and the selenium-GPX4-ferroptosis axis plays a principal role in the regulation of homeostasis of TFH cells (183).

Effector B cells can be divided into BE1 and BE2 lymphocytes from the perspective of cytokine secretion. Thereof, BE1 lymphocytes can generate IFN-γ to promote the transition between Th0 cells and Th1 cells, while BE2 lymphocytes can produce IL-4 to induce Th0 cells differentiation to Th2. Hence, the regulation of B cells by ferroptosis and autophagy may coordinate the immune system in asthma according to modulating the differentiation of T cells. IL-4 as a cytokine of crucial effector Th2 in allergic asthma can also induce autophagy in B cells dependent on JAK signaling *via* an mTOR-independent, PtdIns3K-dependent pathway, which can aggravate asthma through multiple mechanisms (**Figure 7**) (184).

Erastin, a ferroptosis activator, has been proven to induce lipid peroxidation to down-regulate members of the bone morphogenetic protein (BMP) family and promote the differentiation of peripheral blood mononuclear cells to B cells (185). This provides us with a direction: can the number of B cells also be regulated, and how can we use this mechanism to control and alter the sensitivity of B cells to autophagy or ferroptosis (maybe facilitating differentiation of B cells to B1 cells for enhancing ferroptosis sensitivity or inducing effector B lymphocytes into BE1 cells to decrease autophagy inducing asthma exacerbation).

In B cells, IL-4 can induce autophagy dependent on JAK signaling. The enhanced ferroptosis sensitivity resulted from the higher expression of CD36 lead to the regulators targeting GPX4 easily inducing antibody response of B1 cells and MZB cells.

In neutrophils, NETs formation has a tight connection with peptidyl arginine deiminase 4 (PADI4) and NOX. The PI3K–AKT–mTOR axis is a bridge connecting NET induction and autophagy.

Autophagy plays a critical role in the degranulation of mast cells.

### 4.4 The crosslinks and other cells

In asthma, varieties of cells directly result in abnormal symptoms and syndromes by inducing pathophysiologic and histopathologic modification. For example, both eosinophils and mast cells can enhance the permeability of blood vessels. Mast cells contribute to airway remodeling and bronchoconstriction. Eosinophils, neutrophils, basophils and mast cells play an essential role in asthmatic inflammation (**Figure 3**). It is necessary for relieving discomfort and improving prognosis in asthma patients to explore the role of crosslinks between ferroptosis and autophagy plays in these cells.

#### 4.4.1 Eosinophils

Eosinophils play a key role in the eosinophilic inflammatory response. Eosinophils can be recruited to the lung from bone marrow by DCs secreting C-C Motif Chemokine Ligand (CCL)-17 and CCL22. Subsequently, Th2 cells and DCs can be further recruited in asthmatic inflammatory reactions.

The count of eosinophils in BALF positively related with the expression of LC3-II in lung homogenates, indicating that autophagy has a close relationship with the eosinophilic inflammation as well as the severity of asthma. Eosinophilic inflammation has also been shown to alleviate after intranasal treatment with Atg5 shRNA in the context of significantly improved AHR, decreased amount of eosinophils and IL-5 levels in BALF (autophagy can be induced in isolated blood eosinophils in response to IL-5 treatment), and improved histological inflammatory features (186). Meanwhile, eosinophilic inflammation can also be suppressed through ferroptosis-induced agents (FINs) promoting eosinophil death. Furthermore, FIN-induced cellular death can be remarkably attenuated by N-acetylcysteine and GSH (187).

#### 4.4.2 Neutrophils

Neutrophils, as the primary “first line” interaction between primary effector cells and the immune response, gather at the injured position of HEACs from the bone marrow and release chemokines including CXCL-1 and CXCL-8. Neutrophils can also degrade elastin and type-3 collagen (principal components of extracellular matrix) through secreting elastin and proteinase 3, when activated by Th17 cells or Th1 cells. *Meanwhile*, they can fight against pathogens through phagocytosis, degranulation, and neutrophil extracellular traps (NETs). NETs formation has been proven to be upregulated in asthma and has a tight connection with peptidyl arginine deiminase 4 (PADI4) and NOX (**Figure 7**) (188, 189). Both ferroptosis and autophagy can affect the formation of NETs. Stage 3 of NET vacuolization can be influenced by autophagy due to the involvement in the externalization of cytosolic and membrane-bound proteins (190, 191). The PI3K–AKT–mTOR axis is a bridge connecting NET induction and autophagy and has a prominent effect on both (**Figure 7**). Oxidized lipids can promote PADI4-related NETs formation (192). Hence, both autophagy and ferroptosis may control the process of NETs formation to modulate the resistance of neutrophils to pathogens. This idea may efficiently confront the problem of asthmatic therapy, about increasing susceptibility to pathogens due to decreasing anti-inflammatory and antiviral mediators. Autophagy has also been proved to influence the degranulation of neutrophils and ROS production according to NOX and modulate neutrophil-mediated inflammation (193). Ferroptosis may involve the recruitment of neutrophils (194). Therefore, regulation of neutrophilic autophagy and ferroptotic tissue damage may be the approach to alleviate the destruction caused by neutrophilic inflammation (for example cysteinyl leukotrienes [Cys-LTS] and inflammasomes generated by neutrophils can aggravate airway narrowing and promote bronchoconstriction) in asthma.

#### 4.4.3 Mast cells

Mast cells are granulocytic and hematopoietic leukocytes that degranulate to mediate inflammatory responses. In asthma, the activation of mast cells is stimulated by immune or non-immune cells (such as nerve cells) and various cell surface receptors including TLRs, FcϵRI receptors, hormone receptors and cytokine receptors. Autophagy plays a critical role in the degranulation of mast cells (**Figure 7**) (195). If these two forms of RCD can be proved to connect with the regulation of activation of mast cells, it will be reasonable to conclude that the function of mast cells in asthma can be further modulated by ferroptosis and autophagy. The target mutation of GPX4 or GPX4 conditional deletion facilitates instant neuronal death with varieties of ferroptotic characteristics (196). In this way, neuropeptide from nerve cells may be regulated to modulate the activity of mast cells.

When the mechanism of how ferroptosis and autophagy influence the stimulation or dysfunction of the basophils is better known, airway remodeling and asthmatic airway inflammation can be better controlled.

The increased expression of autophagy markers is linked to the increased accumulation of ASM mass. ferroptosis participate in the pathogenesis of airway mass in airway remodeling.

#### 4.4.4 ASM cells

**Figure 8:**
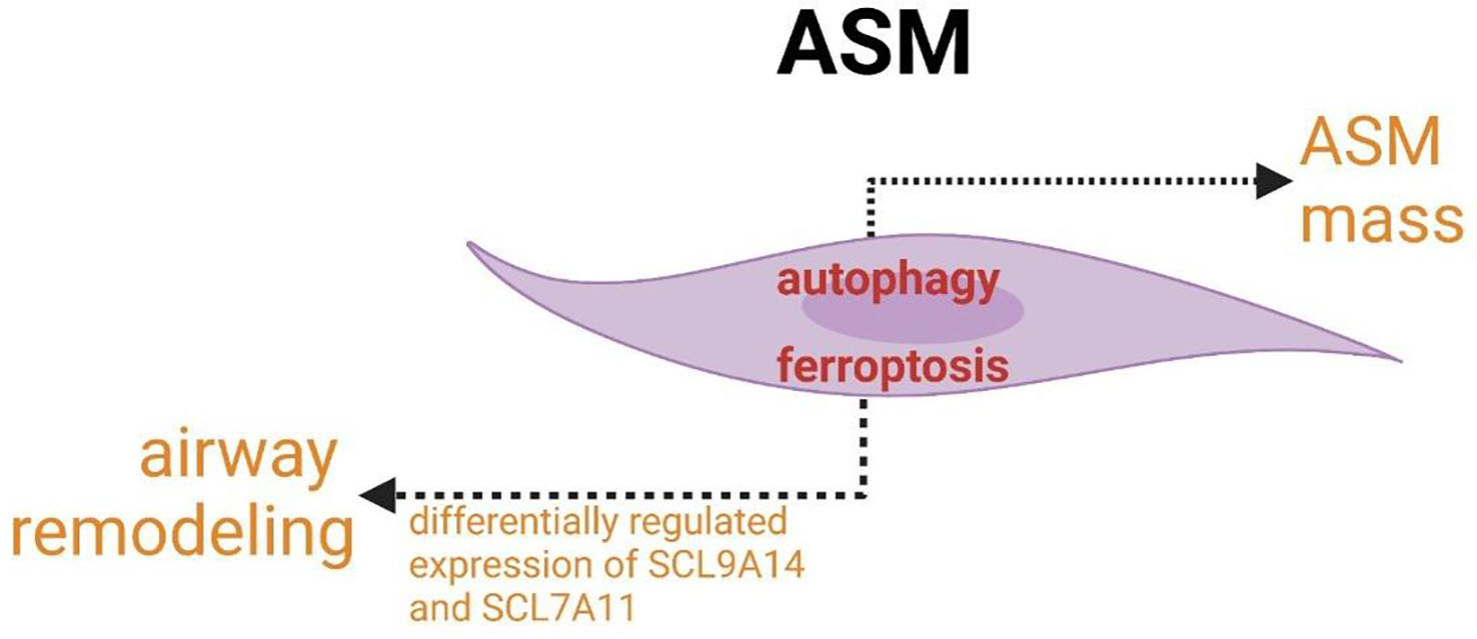
Crosslinks between ferroptosis and autophagy in ASM cells.

ASM is involved not only in airflow obstruction but also in airway inflammation in asthma. The role of crosslinks between autophagy and ferroptosis in abnormal function (increased contractility/decreased relaxation) of ASM cells or the regulation of the size and number may provide new targets for asthmatic therapy and create the new idea for studying the modulatory network of ASM pathogenesis in asthma. The concomitant expression and association of autophagy with airway modeling have been found. ASM mass is increased in asthma and correlates with poor lung function and increased airway responsiveness to multiple contractile agonists, pollens, allergens and so forth. The increased expression of autophagy markers linked to the increased accumulation of ASM mass confirmed that autophagy plays an indispensable role in airway remodeling (**Figure 8**) (197). In addition, autophagy has been shown to be a necessary mechanism for changing the phenotype of HAECs to mesenchymal cells (198, 199). In addition to autophagy, ferroptosis may also participate in the pathogenesis of airway mass in airway remodeling with the differentially regulated expression of SCL9A14 and SCL7A11 (**Figure 8**) (200, 201). Furthermore, key enzymes of PUFAs biosynthesis, the major enzymes correlated with lipid peroxidation and principal proteins related to iron accumulation have been reported to co-express differentially in asthmatic ASM cells (200, 201). Studies of these associated changes may guide us to identify new asthma biomarkers and targets and novel cross-linked regulatory networks to better treat exacerbations of asthma resulting from ASM-inducing airway remodeling, bronchoconstriction and inflammation.

## 5 Conclusion

Both ferroptosis and autophagy are involved in various diseases. The role of these two forms of RCD in treatment has expanded drastically with novel mechanistic details about the regulation and molecular crosstalk between pathways, and the crosslinked effects with immunity and inflammation, which are all previously viewed as independent emerging as topics of particular interest. In this review, we summarize the mechanism of ferroptosis and autophagy and demonstrate the mutual regulation and influence of both of them. Reliance on the common signal pathways and molecules provides multiple potential therapeutic targets. However, the complicated mechanisms of ferroptosis and autophagy make some conclusions contradictory. For example, in macrophages, the suppression of mTOR pathway can activate NF-κB signal pathway, which can enhance the progress of autophagy. But the autophagy induced by TLR2 suppressed NF-κB activation. In DCs, IFN-γ may promote inflammation through ferroptosis, however, suppress the generation of inflammatory cytokines through autophagy. These contradictory mechanisms need to be further explored.

In general, for asthma patients, the deepened cognition of common characteristics of ferroptosis and autophagy can be beneficial based on the modulation of target cells or molecules in the direction of equilibrium. This makes it necessary to clarify the exactly crosslinked molecular events and accurate crosslinked effects with the immunity and inflammation, as well as the clarified upstream and downstream mechanisms when autophagy and ferroptosis trigger cell death or we use the crosslinks between ferroptosis and autophagy to implement a therapeutic schedule. Moreover, to improve the prevention, diagnosis, treatment and prognosis of asthma, researchers should explore more about the crosslinks between ferroptosis and autophagy in asthma-related cells and utilize this accumulated knowledge by refining the relationship between immune homeostasis, regulatory mechanisms, inflammation alleviation and symptom relief. If more specific markers of crosslinked pathways can be discovered and relevant key molecular modulators or novel regulatory approaches based on definite mediators performed by immune cells and signals can be confirmed to be effective in asthmatic therapy, the crosslinks between ferroptosis and autophagy and the role in asthma can be further understood, and the regulation of the crosslinked pathways from perspective of cells and molecules will bring the great therapeutic potential for asthma patients.

## Author contributions

XL and WT wrote the original draft manuscript. JQ, WW, JD and YW reviewed and edited the manuscript. JD and YW supervised the manuscript. All authors contributed to the article and approved the submitted version.

## Glossary

**Table:**

AP	autophagosome
ASM	airway smooth muscle
AHR	airway hyperresponsiveness
AMPK	5′-AMP-activated protein kinase
ARNTL/BMAL1	aryl hydrocarbon receptor nuclear translocator-like
Als	autolysosomes
ATG	autophagy-related gene
CMA	chaperon-mediated autophagy
cDCs	conventional dendritic cells
Cys	cysteine
HMOX1	heme oxygenase 1
GCL	glutamate-cysteine ligase
GPX4	glutathione peroxidase 4
GSS	glutathione synthetase
HAECs	human airway epithelial cells
HIF1	hypoxia-inducible factor 1
Hsp	heat shock protein
ILC2 cells	innate lymphoid type-2 cells
LDs	lipid droplets
LAMP2A	lysosome-associated membrane protein type 2A
mTORC1	mTOR complex 1
MZB lymphocytes	marginal zone B lymphocytes
NETs	neutrophil extracellular traps
NF-κb	nuclear factor-κB
NOD1	nucleotide-binding oligomerization domain-containing protein 1
NOX1	NADPH oxidase 1
NCOA4	nuclear receptor coactivator 4
NKT cells	natural killer T cells
PAMPs	pathogen-associated molecular patterns
PCBPs	poly (RC)- binding proteins (PCBPs) poly (RC)- binding proteins
PLOH	phospholipid alcohol
PUFAs	polyunsaturated fatty acids
RCD	regulated cell death
SQSTM1/P62	Sequestosome 1
SLC11A2	solute carrier family 11 member 2
ULK complex	unc-51-like kinase complex
VPS34	vacuolar protein sorting 34

## References

[1] Zheng M Williams EP Malireddi RS Karki R Banoth B Burton A. Impaired NLRP3 inflammasome activation/pyroptosis leads to robust inflammatory cell death *via* caspase-8/RIPK3 during coronavirus infection. J Biol Chem (2020) 295(41):14040–52. doi: 10.1074/jbc.RA120.015036 PMC7549031 32763970

[2] Sarhan J Liu BC Muendlein HI Li P Nilson R Tang AY. Caspase-8 induces cleavage of gasdermin d to elicit pyroptosis during yersinia infection. Proc Natl Acad Sci (2018) 115(46):E10888–E97. doi: 10.1073/pnas.1809548115 PMC6243247 30381458

[3] Malireddi R Gurung P Kesavardhana S Samir P Burton A Mummareddy H. Innate immune priming in the absence of TAK1 drives RIPK1 kinase activity–independent pyroptosis, apoptosis, necroptosis, and inflammatory disease. J Exp Med (2020) 217(3). doi: 10.1084/jem.20191644 PMC7062518 31869420

[4] Kesavardhana S Malireddi RS Burton AR Porter SN Vogel P Pruett-Miller SM. The Zα2 domain of ZBP1 is a molecular switch regulating influenza-induced PANoptosis and perinatal lethality during development. J Biol Chem (2020) 295(24):8325–30. doi: 10.1074/jbc.RA120.013752 PMC7294087 32350114

[5] Backer JM. The intricate regulation and complex functions of the class III phosphoinositide 3-kinase Vps34. Biochem J (2016) 473(15):2251–71. doi: 10.1042/BCJ20160170 27470591

[6] Ohashi Y Tremel S Williams RL. VPS34 complexes from a structural perspective. J Lipid Res (2019) 60(2):229–41. doi: 10.1194/jlr.R089490 PMC6358306 30397185

[7] Lahiri V Hawkins WD Klionsky DJ. Watch what you (self-) eat: autophagic mechanisms that modulate metabolism. Cell Metab (2019) 29(4):803–26. doi: 10.1016/j.cmet.2019.03.003 PMC6450419 30943392

[8] Ktistakis NT Tooze SA. Digesting the expanding mechanisms of autophagy. Trends Cell Biol (2016) 26(8):624–35. doi: 10.1016/j.tcb.2016.03.006 27050762

[9] Gatica D Lahiri V Klionsky DJ. Cargo recognition and degradation by selective autophagy. Nat Cell Biol (2018) 20(3):233–42. doi: 10.1038/s41556-018-0037-z PMC6028034 29476151

[10] Tanida I Ueno T Kominami E. LC3 conjugation system in mammalian autophagy. Int J Biochem Cell Biol (2004) 36(12):2503–18. doi: 10.1016/j.biocel.2004.05.009 PMC7129593 15325588

[11] Lamark T Kirkin V Dikic I Johansen T. NBR1 and p62 as cargo receptors for selective autophagy of ubiquitinated targets. Cell Cycle (Georgetown Tex) (2009) 8(13):1986–90. doi: 10.4161/cc.8.13.8892 19502794

[12] Shibutani ST Yoshimori T. A current perspective of autophagosome biogenesis. Cell Res (2014) 24(1):58–68. doi: 10.1038/cr.2013.159 24296784 PMC3879706

[13] Orsi A Razi M Dooley H Robinson D Weston A Collinson L. Dynamic and transient interactions of Atg9 with autophagosomes, but not membrane integration, are required for autophagy. Mol Biol Cell (2012) 23(10):1860–73. doi: 10.1091/mbc.e11-09-0746 PMC3350551 22456507

[14] Wang Y Li L Hou C Lai Y Long J Liu J. SNARE-mediated membrane fusion in autophagy. Semin Cell Dev Biol (2016) 60:97–104. doi: 10.1016/j.semcdb.2016.07.009 PMC5161566 27422330

[15] Zhao YG Codogno P Zhang H. Machinery, regulation and pathophysiological implications of autophagosome maturation. Nat Rev Mol Cell Biol (2021) 22(11):733–50. doi: 10.1038/s41580-021-00392-4 PMC8300085 34302147

[16] Gao M Monian P Pan Q Zhang W Xiang J Jiang X. Ferroptosis is an autophagic cell death process. Cell Res (2016) 26(9):1021–32. doi: 10.1038/cr.2016.95 PMC5034113 27514700

[17] Hou W Xie Y Song X Sun X Lotze MT Zeh HJ III. Autophagy promotes ferroptosis by degradation of ferritin. Autophagy (2016) 12(8):1425–8. doi: 10.1080/15548627.2016.1187366 PMC4968231 27245739

[18] Lomia M Tchelidze T Pruidze M. Bronchial asthma as neurogenic paroxysmal inflammatory disease: a randomized trial with carbamazepine. Respir Med (2006) 100(11):1988–96. doi: 10.1016/j.rmed.2006.02.018 16597501

[19] Schiebler M Brown K Hegyi K Newton SM Renna M Hepburn L. Functional drug screening reveals anticonvulsants as enhancers of mTOR-independent autophagic killing of mycobacterium tuberculosis through inositol depletion. EMBO Mol Med (2015) 7(2):127–39. doi: 10.15252/emmm.201404137 PMC4328644 25535254

[20] Ono E Mita H Taniguchi M Higashi N Hasegawa M Miyazaki E. Concentration of 14, 15-leukotriene C4 (eoxin C4) in bronchoalveolar lavage fluid. Clin Exp Allergy (2009) 39(9):1348–52. doi: 10.1111/j.1365-2222.2009.03261.x 19438588

[21] Hajek AR Lindley AR Favoreto S Carter R Schleimer RP Kuperman DA. 12/15-lipoxygenase deficiency protects mice from allergic airways inflammation and increases secretory IgA levels. J Allergy Clin Immunol (2008) 122(3):633–9. e3. doi: 10.1016/j.jaci.2008.06.021 18692885 PMC2802267

[22] Zhao J Dar HH Deng Y St. Croix CM Li Z Minami Y. PEBP1 acts as a rheostat between prosurvival autophagy and ferroptotic death in asthmatic epithelial cells. Proc Natl Acad Sci (2020) 117(25):14376–85. doi: 10.1073/pnas.1921618117 PMC7321965 32513718

[23] Hubbard VM Valdor R Macian F Cuervo AM. Selective autophagy in the maintenance of cellular homeostasis in aging organisms. Biogerontology (2012) 13:21–35. doi: 10.1007/s10522-011-9331-x 21461872

[24] Zhang Z Yao Z Wang L Ding H Shao J Chen A. Activation of ferritinophagy is required for the RNA-binding protein ELAVL1/HuR to regulate ferroptosis in hepatic stellate cells. Autophagy (2018) 14(12):2083–103. doi: 10.1080/15548627.2018.1503146 PMC6984765 30081711

[25] Park E Chung SW. ROS-mediated autophagy increases intracellular iron levels and ferroptosis by ferritin and transferrin receptor regulation. Cell Death Disease (2019) 10(11):1–10. doi: 10.1038/s41419-019-2064-5 PMC6817894 31659150

[26] Protchenko O Baratz E Jadhav S Li F Shakoury-Elizeh M Gavrilova O. Iron chaperone poly rC binding protein 1 protects mouse liver from lipid peroxidation and steatosis. Hepatology (2021) 73(3):1176–93. doi: 10.1002/hep.31328 PMC8364740 32438524

[27] Wang Y-Q Chang S-Y Wu Q Gou Y-J Jia L Cui Y-M. The protective role of mitochondrial ferritin on erastin-induced ferroptosis. Front Aging Neurosci (2016) 8:308. doi: 10.3389/fnagi.2016.00308 28066232 PMC5167726

[28] Bailey AP Koster G Guillermier C Hirst EM MacRae JI Lechene CP. Antioxidant role for lipid droplets in a stem cell niche of drosophila. Cell (2015) 163(2):340–53. doi: 10.1016/j.cell.2015.09.020 PMC4601084 26451484

[29] Bai Y Meng L Han L Jia Y Zhao Y Gao H. Lipid storage and lipophagy regulates ferroptosis. Biochem Biophys Res Commun (2019) 508(4):997–1003. doi: 10.1016/j.bbrc.2018.12.039 30545638

[30] Yang M Chen P Liu J Zhu S Kroemer G Klionsky DJ. Clockophagy is a novel selective autophagy process favoring ferroptosis. Sci Adv (2019) 5(7):eaaw2238. doi: 10.1126/sciadv.aaw2238 31355331 PMC6656546

[31] Dice JF. Chaperone-mediated autophagy. Autophagy (2007) 3(4):295–9. doi: 10.4161/auto.4144 17404494

[32] Wu Z Geng Y Lu X Shi Y Wu G Zhang M. Chaperone-mediated autophagy is involved in the execution of ferroptosis. Proc Natl Acad Sci (2019) 116(8):2996–3005. doi: 10.1073/pnas.1819728116 30718432 PMC6386716

[33] Ingold I Berndt C Schmitt S Doll S Poschmann G Buday K. Selenium utilization by GPX4 is required to prevent hydroperoxide-induced ferroptosis. Cell (2018) 172(3):409–22. e21. doi: 10.1016/j.cell.2017.11.048 29290465

[34] Ursini F Maiorino M. Lipid peroxidation and ferroptosis: the role of GSH and GPx4. Free Radical Biol Med (2020) 152:175–85. doi: 10.1016/j.freeradbiomed.2020.02.027 32165281

[35] Song X Zhu S Chen P Hou W Wen Q Liu J. AMPK-mediated BECN1 phosphorylation promotes ferroptosis by directly blocking system xc–activity. Curr Biol (2018) 28(15):2388–99. e5. doi: 10.1016/j.cub.2018.05.094 30057310 PMC6081251

[36] Shen M Li Y Wang Y Shao J Zhang F Yin G. N6-methyladenosine modification regulates ferroptosis through autophagy signaling pathway in hepatic stellate cells. Redox Biol (2021) 47:102151. doi: 10.1016/j.redox.2021.102151 34607160 PMC8495178

[37] Ishimoto T Nagano O Yae T Tamada M Motohara T Oshima H. CD44 variant regulates redox status in cancer cells by stabilizing the xCT subunit of system xc– and thereby promotes tumor growth. Cancer Cell (2011) 19(3):387–400. doi: 10.1016/j.ccr.2011.01.038 21397861

[38] Hayano M Yang W Corn C Pagano N Stockwell B. Loss of cysteinyl-tRNA synthetase (CARS) induces the transsulfuration pathway and inhibits ferroptosis induced by cystine deprivation. Cell Death Differentiation (2016) 23(2):270–8. doi: 10.1038/cdd.2015.93 PMC4716307 26184909

[39] Wang L Cai H Hu Y Liu F Huang S Zhou Y. A pharmacological probe identifies cystathionine β-synthase as a new negative regulator for ferroptosis. Cell Death disease (2018) 9(10):1–17. doi: 10.1038/s41419-018-1063-2 30258181 PMC6158189

[40] Liu J Kuang F Kroemer G Klionsky DJ Kang R Tang D. Autophagy-dependent ferroptosis: machinery and regulation. Cell Chem Biol (2020) 27(4):420–35. doi: 10.1016/j.chembiol.2020.02.005 PMC7166192 32160513

[41] Gao M Yi J Zhu J Minikes AM Monian P Thompson CB. Role of mitochondria in ferroptosis. Mol Cell (2019) 73(2):354–63. e3. doi: 10.1016/j.molcel.2018.10.042 30581146 PMC6338496

[42] Li C Zhang Y Liu J Kang R Klionsky DJ Tang D. Mitochondrial DNA stress triggers autophagy-dependent ferroptotic death. Autophagy (2021) 17(4):948–60. doi: 10.1080/15548627.2020.1739447 PMC8078708 32186434

[43] Lee H Zandkarimi F Zhang Y Meena JK Kim J Zhuang L. Energy-stress-mediated AMPK activation inhibits ferroptosis. Nat Cell Biol (2020) 22(2):225–34. doi: 10.1038/s41556-020-0461-8 PMC7008777 32029897

[44] Basit F Van Oppen LM Schöckel L Bossenbroek HM Van Emst-de Vries SE Hermeling JC. Mitochondrial complex I inhibition triggers a mitophagy-dependent ROS increase leading to necroptosis and ferroptosis in melanoma cells. Cell Death Disease (2017) 8(3):e2716–e. doi: 10.1038/cddis.2017.133 PMC5386536 28358377

[45] Chang L-C Chiang S-K Chen S-E Yu Y-L Chou R-H Chang W-C. Heme oxygenase-1 mediates BAY 11–7085 induced ferroptosis. Cancer letters (2018) 416:124–37. doi: 10.1016/j.canlet.2017.12.025 29274359

[46] Xiong Q Li X Li W Chen G Xiao H Li P. WDR45 mutation impairs the autophagic degradation of transferrin receptor and promotes ferroptosis. Front Mol biosciences (2021) 8:645831. doi: 10.3389/fmolb.2021.645831 PMC8126626 34012978

[47] Peng Y Yang J Li Z Chen S Tang X Zhou J. Overexpression of SLC40A1 inhibits the malignancy of hepatocellular carcinoma MHCC-97H cells by stimulation of autophagy. Biomed Signal Process Control (2022) 75:103554. doi: 10.1016/j.bspc.2022.103554

[48] He H Wang L Qiao Y Yang B Yin D He M. Epigallocatechin-3-gallate pretreatment alleviates doxorubicin-induced ferroptosis and cardiotoxicity by upregulating AMPKα2 and activating adaptive autophagy. Redox Biol (2021) 48:102185. doi: 10.1016/j.redox.2021.102185 34775319 PMC8600154

[49] Haeggstrom JZ Funk CD. Lipoxygenase and leukotriene pathways: biochemistry, biology, and roles in disease. Chem Rev (2011) 111(10):5866–98. doi: 10.1021/cr200246d 21936577

[50] Chen X Song X Li J Zhang R Yu C Zhou Z. Identification of HPCAL1 as a specific autophagy receptor involved in ferroptosis. Autophagy (2022) 19(1):1–21. doi: 10.1080/15548627.2022.2059170 35403545 PMC9809962

[51] Gupta U Ghosh S Wallace CT Shang P Xin Y Nair AP. Increased LCN2 (lipocalin 2) in the RPE decreases autophagy and activates inflammasome-ferroptosis processes in a mouse model of dry AMD. Autophagy (2022) 19(1):1–20. doi: 10.1080/15548627.2022.2062887 35473441 PMC9809950

[52] Liu J Liu Y Wang Y Li C Xie Y Klionsky DJ. TMEM164 is a new determinant of autophagy-dependent ferroptosis. Autophagy (2022) 19(3):1–12. doi: 10.1080/15548627.2022.2111635 35947500 PMC9980451

[53] Ichimura Y Waguri S Sou Y-s Kageyama S Hasegawa J Ishimura R. Phosphorylation of p62 activates the Keap1-Nrf2 pathway during selective autophagy. Mol Cell (2013) 51(5):618–31. doi: 10.1016/j.molcel.2013.08.003 24011591

[54] Peng Q Liu H Luo Z Zhao H Wang X Guan X. Effect of autophagy on ferroptosis in foam cells *via* Nrf2. Mol Cell Biochem (2022) 477(5):1597–606. doi: 10.1007/s11010-021-04347-3 35195807

[55] Zhang Z Guo M Li Y Shen M Kong D Shao J. RNA-Binding protein ZFP36/TTP protects against ferroptosis by regulating autophagy signaling pathway in hepatic stellate cells. Autophagy (2020) 16(8):1482–505. doi: 10.1080/15548627.2019.1687985 PMC7469536 31679460

[56] Dikic I Elazar Z. Mechanism and medical implications of mammalian autophagy. Nat Rev Mol Cell Biol (2018) 19(6):349–64. doi: 10.1038/s41580-018-0003-4 29618831

[57] Tooze SA Yoshimori T. The origin of the autophagosomal membrane. Nat Cell Biol (2010) 12(9):831–5. doi: 10.1038/ncb0910-831 20811355

[58] Xie Y Kang R Sun X Zhong M Huang J Klionsky DJ. Posttranslational modification of autophagy-related proteins in macroautophagy. Autophagy (2015) 11(1):28–45. doi: 10.4161/15548627.2014.984267 25484070 PMC4502723

[59] Nakamura S Yoshimori T. New insights into autophagosome–lysosome fusion. J Cell science (2017) 130(7):1209–16. doi: 10.1242/jcs.196352 28302910

[60] Chen D Rauh M Buchfelder M Eyupoglu IY Savaskan N. The oxido-metabolic driver ATF4 enhances temozolamide chemo-resistance in human gliomas. Oncotarget (2017) 8(31):51164. doi: 10.18632/oncotarget.17737 28881638 PMC5584239

[61] Rahmani M Davis EM Crabtree TR Habibi JR Nguyen TK Dent P. The kinase inhibitor sorafenib induces cell death through a process involving induction of endoplasmic reticulum stress. Mol Cell Biol (2007) 27(15):5499–513. doi: 10.1128/MCB.01080-06 PMC1952105 17548474

[62] Yang C Ma X Wang Z Zeng X Hu Z Ye Z. Curcumin induces apoptosis and protective autophagy in castration-resistant prostate cancer cells through iron chelation. Drug design Dev Ther (2017) 11:431. doi: 10.2147/DDDT.S126964 PMC5317247 28243065

[63] Bhattacharya A Ghosh P Singh A Ghosh A Bhowmick A Sinha DK. Delineating the complex mechanistic interplay between NF-κβ driven mTOR depedent autophagy and monocyte to macrophage differentiation: A functional perspective. Cell signalling (2021) 88:110150. doi: 10.1016/j.cellsig.2021.110150 34547324

[64] Jang EJ Kim DH Lee B Lee EK Chung KW Moon KM. Activation of proinflammatory signaling by 4-hydroxynonenal-Src adducts in aged kidneys. Oncotarget (2016) 7(32):50864. doi: 10.18632/oncotarget.10854 27472463 PMC5239442

[65] Copetti T Bertoli C Dalla E Demarchi F Schneider C. p65/RelA modulates BECN1 transcription and autophagy. Mol Cell Biol (2009) 29(10):2594–608. doi: 10.1128/MCB.01396-08 PMC2682036 19289499

[66] Kang R Chen R Zhang Q Hou W Wu S Cao L. HMGB1 in health and disease. Mol aspects Med (2014) 40:1–116. doi: 10.1016/j.mam.2014.05.001 25010388 PMC4254084

[67] Wen Q Liu J Kang R Zhou B Tang D. The release and activity of HMGB1 in ferroptosis. Biochem Biophys Res Commun (2019) 510(2):278–83. doi: 10.1016/j.bbrc.2019.01.090 30686534

[68] Liu L Yang M Kang R Wang Z Zhao Y Yu Y. DAMP-mediated autophagy contributes to drug resistance. Autophagy (2011) 7(1):112–4. doi: 10.4161/auto.7.1.14005 PMC3039734 21068541

[69] Tasdemir E Maiuri MC Morselli E Criollo A D'Amelio M Djavaheri-Mergny M. A dual role of p53 in the control of autophagy. Autophagy (2008) 4(6):810–4. doi: 10.4161/auto.6486 18604159

[70] Ou Y Wang S-J Li D Chu B Gu W. Activation of SAT1 engages polyamine metabolism with p53-mediated ferroptotic responses. Proc Natl Acad Sci (2016) 113(44):E6806–E12. doi: 10.1073/pnas.1607152113 PMC5098629 27698118

[71] Jiang L Kon N Li T Wang S-J Su T Hibshoosh H. Ferroptosis as a p53-mediated activity during tumour suppression. Nature (2015) 520(7545):57–62. doi: 10.1038/nature14344 25799988 PMC4455927

[72] Zhang W Gai C Ding D Wang F Li W. Targeted p53 on small-molecules-induced ferroptosis in cancers. Front Oncol (2018) 8:507. doi: 10.3389/fonc.2018.00507 30450337 PMC6224449

[73] Xie Y Zhu S Song X Sun X Fan Y Liu J. The tumor suppressor p53 limits ferroptosis by blocking DPP4 activity. Cell Rep (2017) 20(7):1692–704. doi: 10.1016/j.celrep.2017.07.055 28813679

[74] Wang S-J Li D Ou Y Jiang L Chen Y Zhao Y. Acetylation is crucial for p53-mediated ferroptosis and tumor suppression. Cell Rep (2016) 17(2):366–73. doi: 10.1016/j.celrep.2016.09.022 PMC5227654 27705786

[75] Sun M Li J Mao L Wu J Deng Z He M. p53 deacetylation alleviates sepsis-induced acute kidney injury by promoting autophagy. Front Immunol (2021) 12. doi: 10.3389/fimmu.2021.685523 PMC8318785 34335587

[76] Liu Q Wang K. The induction of ferroptosis by impairing STAT3/Nrf2/GPx4 signaling enhances the sensitivity of osteosarcoma cells to cisplatin. Cell Biol Int (2019) 43(11):1245–56. doi: 10.1002/cbin.11121 30811078

[77] Castets P Rion N Théodore M Falcetta D Lin S Reischl M. mTORC1 and PKB/Akt control the muscle response to denervation by regulating autophagy and HDAC4. Nat Commun (2019) 10(1):1–16. doi: 10.1038/s41467-019-11227-4 31320633 PMC6639401

[78] Kang R Tang D Lotze MT Zeh I Herbert J. AGER/RAGE-mediated autophagy promotes pancreatic tumorigenesis and bioenergetics through the IL6-pSTAT3 pathway. Autophagy (2012) 8(6):989–91. doi: 10.4161/auto.20258 PMC3427269 22722139

[79] Zhang Z-B Xiong L-L Xue L-L Deng Y-P Du R-L Hu Q. MiR-127-3p targeting CISD1 regulates autophagy in hypoxic–ischemic cortex. Cell Death disease (2021) 12(3):1–17. doi: 10.1038/s41419-021-03541-x 33723216 PMC7961148

[80] Tamir S Rotem-Bamberger S Katz C Morcos F Hailey KL Zuris JA. Integrated strategy reveals the protein interface between cancer targets bcl-2 and NAF-1. Proc Natl Acad Sci (2014) 111(14):5177–82. doi: 10.1073/pnas.1403770111 PMC3986192 24706857

[81] Sohn Y-S Tamir S Song L Michaeli D Matouk I Conlan AR. NAF-1 and mitoNEET are central to human breast cancer proliferation by maintaining mitochondrial homeostasis and promoting tumor growth. Proc Natl Acad Sci (2013) 110(36):14676–81. doi: 10.1073/pnas.1313198110 PMC3767537 23959881

[82] Yuan H Li X Zhang X Kang R Tang D. CISD1 inhibits ferroptosis by protection against mitochondrial lipid peroxidation. Biochem Biophys Res Commun (2016) 478(2):838–44. doi: 10.1016/j.bbrc.2016.08.034 27510639

[83] Hou J Rao M Zheng W Fan J Law BYK. Advances on cell autophagy and its potential regulatory factors in renal ischemia-reperfusion injury. DNA Cell Biol (2019) 38(9):895–904. doi: 10.1089/dna.2019.4767 31347925

[84] Shen L Qi Z Zhu Y Song X Xuan C Ben P. Phosphorylated heat shock protein 27 promotes lipid clearance in hepatic cells through interacting with STAT3 and activating autophagy. Cell signalling (2016) 28(8):1086–98. doi: 10.1016/j.cellsig.2016.05.008 27185187

[85] Tang D Kang R Livesey KM Kroemer G Billiar TR Van Houten B. High-mobility group box 1 is essential for mitochondrial quality control. Cell Metab (2011) 13(6):701–11. doi: 10.1016/j.cmet.2011.04.008 PMC3293110 21641551

[86] Tang D Kang R Livesey KM Cheh C-W Farkas A Loughran P. Endogenous HMGB1 regulates autophagy. J Cell Biol (2010) 190(5):881–92. doi: 10.1083/jcb.200911078 PMC2935581 20819940

[87] Sun X Ou Z Xie M Kang R Fan Y Niu X. HSPB1 as a novel regulator of ferroptotic cancer cell death. Oncogene (2015) 34(45):5617–25. doi: 10.1038/onc.2015.32 PMC4640181 25728673

[88] Wang Z Li Y Wang D Shen Y. Ferroptosis molecular inducers: A future direction for malignant tumor chemotherapy. Biocell (2022) 46(7):1599. doi: 10.32604/biocell.2022.018530

[89] Song X Xie Y Kang R Hou W Sun X Epperly MW. FANCD2 protects against bone marrow injury from ferroptosis. Biochem Biophys Res Commun (2016) 480(3):443–9. doi: 10.1016/j.bbrc.2016.10.068 PMC6591579 27773819

[90] Sumpter R Sirasanagandla S Fernández ÁF Wei Y Dong X Franco L. Fanconi anemia proteins function in mitophagy and immunity. Cell (2016) 165(4):867–81. doi: 10.1016/j.cell.2016.04.006 PMC4881391 27133164

[91] Li Y Zhang L Zhou J Luo S Huang R Zhao C. Nedd4 E3 ubiquitin ligase promotes cell proliferation and autophagy. Cell proliferation (2015) 48(3):338–47. doi: 10.1111/cpr.12184 PMC6495840 25809873

[92] Yang Y Luo M Zhang K Zhang J Gao T Connell DO. Nedd4 ubiquitylates VDAC2/3 to suppress erastin-induced ferroptosis in melanoma. Nat Commun (2020) 11(1):1–14. doi: 10.1038/s41467-020-14324-x 31974380 PMC6978386

[93] Wang Y Liu Y Liu J Kang R Tang D. NEDD4L-mediated LTF protein degradation limits ferroptosis. Biochem Biophys Res Commun (2020) 531(4):581–7. doi: 10.1016/j.bbrc.2020.07.032 32811647

[94] Halim TY Steer CA Mathä L Gold MJ Martinez-Gonzalez I McNagny KM. Group 2 innate lymphoid cells are critical for the initiation of adaptive T helper 2 cell-mediated allergic lung inflammation. Immunity (2014) 40(3):425–35. doi: 10.1016/j.immuni.2014.01.011 PMC4210641 24613091

[95] Willart MA Deswarte K Pouliot P Braun H Beyaert R Lambrecht BN. Interleukin-1α controls allergic sensitization to inhaled house dust mite *via* the epithelial release of GM-CSF and IL-33. J Exp Med (2012) 209(8):1505–17. doi: 10.1084/jem.20112691 PMC3409497 22802353

[96] Besnard AG Togbe D Guillou N Erard F Quesniaux V Ryffel B. IL-33-activated dendritic cells are critical for allergic airway inflammation. Eur J Immunol (2011) 41(6):1675–86. doi: 10.1002/eji.201041033 21469105

[97] Bell BD Kitajima M Larson RP Stoklasek TA Dang K Sakamoto K. The transcription factor STAT5 is critical in dendritic cells for the development of TH2 but not TH1 responses. Nat Immunol (2013) 14(4):364–71. doi: 10.1038/ni.2541 PMC4161284 23435120

[98] Chu DK Llop-Guevara A Walker TD Flader K Goncharova S Boudreau JE. IL-33, but not thymic stromal lymphopoietin or IL-25, is central to mite and peanut allergic sensitization. J Allergy Clin Immunol (2013) 131(1):187–200. e8. doi: 10.1016/j.jaci.2012.08.002 23006545

[99] Fahy JV. Type 2 inflammation in asthma–present in most, absent in many. Nat Rev Immunol (2015) 15(1):57–65. doi: 10.1038/nri3786 25534623 PMC4390063

[100] Crosby LM Waters CM. Epithelial repair mechanisms in the lung. Am J Physiol-Lung Cell Mol Physiol (2010) 298(6):L715–L31. doi: 10.1152/ajplung.00361.2009 PMC2886606 20363851

[101] Wenzel SE. Asthma phenotypes: the evolution from clinical to molecular approaches. Nat Med (2012) 18(5):716–25. doi: 10.1038/nm.2678 22561835

[102] Dickinson JD Alevy Y Malvin NP Patel KK Gunsten SP Holtzman MJ. IL13 activates autophagy to regulate secretion in airway epithelial cells. Autophagy (2016) 12(2):397–409. doi: 10.1080/15548627.2015.1056967 26062017 PMC4835964

[103] Dickinson JD Sweeter JM Warren KJ Ahmad IM De Deken X Zimmerman MC. Autophagy regulates DUOX1 localization and superoxide production in airway epithelial cells during chronic IL-13 stimulation. Redox Biol (2018) 14:272–84. doi: 10.1016/j.redox.2017.09.013 PMC5635347 28982074

[104] Wong CK Hu S Leung KM-L Dong J He L Chu YJ. NOD-like receptors mediated activation of eosinophils interacting with bronchial epithelial cells: a link between innate immunity and allergic asthma. Cell Mol Immunol (2013) 10(4):317–29. doi: 10.1038/cmi.2012.77 PMC4003204 23524653

[105] Martinon F Mayor A Tschopp J. The inflammasomes: guardians of the body. Annu Rev Immunol (2009) 27:229–65. doi: 10.1146/annurev.immunol.021908.132715 19302040

[106] Travassos LH Carneiro LA Ramjeet M Hussey S Kim Y-G Magalhães JG. Nod1 and Nod2 direct autophagy by recruiting ATG16L1 to the plasma membrane at the site of bacterial entry. Nat Immunol (2010) 11(1):55–62. doi: 10.1038/ni.1823 19898471

[107] Fernández-García V González-Ramos S Avendaño-Ortiz J Martín-Sanz P Delgado C Castrillo A. NOD1 splenic activation confers ferroptosis protection and reduces macrophage recruitment under pro-atherogenic conditions. Biomed Pharmacother (2022) 148:112769. doi: 10.1016/j.biopha.2022.112769 35247718

[108] Klöditz K Fadeel B. Three cell deaths and a funeral: macrophage clearance of cells undergoing distinct modes of cell death. Cell Death discovery (2019) 5(1):1–9. doi: 10.1038/s41420-019-0146-x PMC6368547 30774993

[109] Levine B Mizushima N Virgin HW. Autophagy in immunity and inflammation. Nature (2011) 469(7330):323–35. doi: 10.1038/nature09782 PMC3131688 21248839

[110] Murai H Okazaki S Hayashi H Kawakita A Hosoki K Yasutomi M. Alternaria extract activates autophagy that induces IL-18 release from airway epithelial cells. Biochem Biophys Res Commun (2015) 464(4):969–74. doi: 10.1016/j.bbrc.2015.05.076 PMC4915742 26032499

[111] Li W Wu Y Zhao Y Li Z Chen H Dong L. MTOR suppresses autophagy-mediated production of IL25 in allergic airway inflammation. Thorax (2020) 75(12):1047–57. doi: 10.1136/thoraxjnl-2019-213771 33077617

[112] Holgate ST. Innate and adaptive immune responses in asthma. Nat Med (2012) 18(5):673–83. doi: 10.1038/nm.2731 22561831

[113] Worbs T Hammerschmidt SI Förster R. Dendritic cell migration in health and disease. Nat Rev Immunol (2017) 17(1):30–48. doi: 10.1038/nri.2016.116 27890914

[114] Izumi G Nakano H Nakano K Whitehead GS Grimm SA Fessler MB. CD11b+ lung dendritic cells at different stages of maturation induce Th17 or Th2 differentiation. Nat Commun (2021) 12(1):5029. doi: 10.1038/s41467-021-25307-x 34413303 PMC8377117

[115] Lambrecht BN Hammad H. The immunology of asthma. Nat Immunol (2015) 16(1):45–56. doi: 10.1038/ni.3049 25521684

[116] Ghislat G Lawrence T. Autophagy in dendritic cells. Cell Mol Immunol (2018) 15(11):944–52. doi: 10.1038/cmi.2018.2 PMC6207777 29578531

[117] Chen X Xu S Zhao C Liu B. Role of TLR4/NADPH oxidase 4 pathway in promoting cell death through autophagy and ferroptosis during heart failure. Biochem Biophys Res Commun (2019) 516(1):37–43. doi: 10.1016/j.bbrc.2019.06.015 31196626

[118] Terawaki S Camosseto V Prete F Wenger T Papadopoulos A Rondeau C. RUN and FYVE domain–containing protein 4 enhances autophagy and lysosome tethering in response to interleukin-4. J Cell Biol (2015) 210(7):1133–52. doi: 10.1083/jcb.201501059 PMC4586740 26416964

[119] Khan N Vidyarthi A Pahari S Negi S Aqdas M Nadeem S. Signaling through NOD-2 and TLR-4 bolsters the T cell priming capability of dendritic cells by inducing autophagy. Sci Rep (2016) 6(1):1–8. doi: 10.1038/srep19084 26754352 PMC4709561

[120] Wildenberg M Koelink P Diederen K Te Velde A Wolfkamp S Nuij V. The ATG16L1 risk allele associated with crohn’s disease results in a Rac1-dependent defect in dendritic cell migration that is corrected by thiopurines. Mucosal Immunol (2017) 10(2):352–60. doi: 10.1038/mi.2016.65 27435106

[121] Ishikawa H Barber GN. STING is an endoplasmic reticulum adaptor that facilitates innate immune signalling. Nature (2008) 455(7213):674–8. doi: 10.1038/nature07317 PMC2804933 18724357

[122] Barber GN. STING: infection, inflammation and cancer. Nat Rev Immunol (2015) 15(12):760–70. doi: 10.1038/nri3921 PMC5004891 26603901

[123] Motwani M Pesiridis S Fitzgerald KA. DNA Sensing by the cGAS–STING pathway in health and disease. Nat Rev Genet (2019) 20(11):657–74. doi: 10.1038/s41576-019-0151-1 31358977

[124] Zhang R Kang R Tang D. The STING1 network regulates autophagy and cell death. Signal Transduction Targeted Ther (2021) 6(1):1–13. doi: 10.1038/s41392-021-00613-4 PMC8172903 34078874

[125] Zhou D Li P Lin Y Lott JM Hislop AD Canaday DH. Lamp-2a facilitates MHC class II presentation of cytoplasmic antigens. Immunity (2005) 22(5):571–81. doi: 10.1016/j.immuni.2005.03.009 15894275

[126] Wiernicki B Maschalidi S Pinney J Adjemian S Vanden Berghe T Ravichandran KS. Cancer cells dying from ferroptosis impede dendritic cell-mediated anti-tumor immunity. Nat Commun (2022) 13(1):1–15. doi: 10.1038/s41467-022-31218-2 35760796 PMC9237053

[127] Thurston TL Wandel MP von Muhlinen N Foeglein Á Randow F. Galectin 8 targets damaged vesicles for autophagy to defend cells against bacterial invasion. Nature (2012) 482(7385):414–8. doi: 10.1038/nature10744 PMC3343631 22246324

[128] Fujita N Morita E Itoh T Tanaka A Nakaoka M Osada Y. Recruitment of the autophagic machinery to endosomes during infection is mediated by ubiquitin. J Cell Biol (2013) 203(1):115–28. doi: 10.1083/jcb.201304188 PMC3798248 24100292

[129] Brooks CR Yeung MY Brooks YS Chen H Ichimura T Henderson JM. KIM-1-/TIM-1-mediated phagocytosis links ATG 5-/ULK 1-dependent clearance of apoptotic cells to antigen presentation. EMBO J (2015) 34(19):2441–64. doi: 10.15252/embj.201489838 PMC4601664 26282792

[130] MacMicking JD. Interferon-inducible effector mechanisms in cell-autonomous immunity. Nat Rev Immunol (2012) 12(5):367–82. doi: 10.1038/nri3210 PMC4150610 22531325

[131] Lee Y Sasai M Ma JS Sakaguchi N Ohshima J Bando H. p62 plays a specific role in interferon-γ-induced presentation of a toxoplasma vacuolar antigen. Cell Rep (2015) 13(2):223–33. doi: 10.1016/j.celrep.2015.09.005 26440898

[132] Zang F Chen Y Lin Z Cai Z Yu L Xu F. Autophagy is involved in regulating the immune response of dendritic cells to influenza a (H1N1) pdm09 infection. Immunology (2016) 148(1):56–69. doi: 10.1111/imm.12587 26800655 PMC4819145

[133] Sato H Fujiwara K Sagara J-i Bannai S. Induction of cystine transport activity in mouse peritoneal macrophages by bacterial lipopolysaccharide. Biochem J (1995) 310(2):547–51. doi: 10.1042/bj3100547 PMC1135929 7654193

[134] Li G Liang X Lotze MT. HMGB1: the central cytokine for all lymphoid cells. Front Immunol (2013) 4:68. doi: 10.3389/fimmu.2013.00068 23519706 PMC3602962

[135] Gordon S Taylor PR. Monocyte and macrophage heterogeneity. Nat Rev Immunol (2005) 5(12):953–64. doi: 10.1038/nri1733 16322748

[136] Gordon S Martinez FO. Alternative activation of macrophages: mechanism and functions. Immunity (2010) 32(5):593–604. doi: 10.1016/j.immuni.2010.05.007 20510870

[137] Murray PJ Allen JE Biswas SK Fisher EA Gilroy DW Goerdt S. Macrophage activation and polarization: nomenclature and experimental guidelines. Immunity (2014) 41(1):14–20. doi: 10.1016/j.immuni.2014.06.008 25035950 PMC4123412

[138] Handa P Thomas S Morgan-Stevenson V Maliken BD Gochanour E Boukhar S. Iron alters macrophage polarization status and leads to steatohepatitis and fibrogenesis. J Leukocyte Biol (2019) 105(5):1015–26. doi: 10.1002/JLB.3A0318-108R 30835899

[139] Zhou Y Que KT Zhang Z Yi ZJ Zhao PX You Y. Iron overloaded polarizes macrophage to proinflammation phenotype through ROS/acetyl-p53 pathway. Cancer Med (2018) 7(8):4012–22. doi: 10.1002/cam4.1670 PMC6089144 29989329

[140] Chen W Ma T Shen X-n Xia X-f Xu G-d Bai X-l. Macrophage-induced tumor angiogenesis is regulated by the TSC2–mTOR PathwayTSC2–mTOR regulates macrophage-induced tumor angiogenesis. Cancer Res (2012) 72(6):1363–72. doi: 10.1158/0008-5472.CAN-11-2684 22287548

[141] Schlaepfer E Rochat M-A Duo L Speck RF. Triggering TLR2,-3,-4,-5, and-8 reinforces the restrictive nature of M1-and M2-polarized macrophages to HIV. J virol (2014) 88(17):9769–81. doi: 10.1128/JVI.01053-14 PMC4136350 24942590

[142] Biswas SK Lewis CE. NF-κB as a central regulator of macrophage function in tumors. J leukocyte Biol (2010) 88(5):877–84. doi: 10.1189/jlb.0310153 20573802

[143] Chang C-P Su Y-C Lee P-H Lei H-Y. Targeting NFKB by autophagy to polarize hepatoma-associated macrophage differentiation. Autophagy (2013) 9(4):619–21. doi: 10.4161/auto.23546 PMC3627680 23360732

[144] Chang C Su Y Hu C Lei H. TLR2-dependent selective autophagy regulates NF-κB lysosomal degradation in hepatoma-derived M2 macrophage differentiation. Cell Death Differentiation (2013) 20(3):515–23. doi: 10.1038/cdd.2012.146 PMC3569990 23175187

[145] Roca H Varsos ZS Sud S Craig MJ Ying C Pienta KJ. CCL2 and interleukin-6 promote survival of human CD11b+ peripheral blood mononuclear cells and induce M2-type macrophage polarization. J Biol Chem (2009) 284(49):34342–54. doi: 10.1074/jbc.M109.042671 PMC2797202 19833726

[146] Djudjaj S Martin IV Buhl EM Nothofer NJ Leng L Piecychna M. Macrophage migration inhibitory factor limits renal inflammation and fibrosis by counteracting tubular cell cycle arrest. J Am Soc Nephrol (2017) 28(12):3590–604. doi: 10.1681/ASN.2017020190 PMC5698074 28801314

[147] Lv L-L Feng Y Wen Y Wu W-J Ni H-F Li Z-L. Exosomal CCL2 from tubular epithelial cells is critical for albumin-induced tubulointerstitial inflammation. J Am Soc Nephrol (2018) 29(3):919–35. doi: 10.1681/ASN.2017050523 PMC5827595 29295871

[148] Wang Y Quan F Cao Q Lin Y Yue C Bi R. Quercetin alleviates acute kidney injury by inhibiting ferroptosis. J advanced Res (2021) 28:231–43. doi: 10.1016/j.jare.2020.07.007 PMC7753233 33364059

[149] Dai E Han L Liu J Xie Y Kroemer G Klionsky DJ. Autophagy-dependent ferroptosis drives tumor-associated macrophage polarization *via* release and uptake of oncogenic KRAS protein. Autophagy (2020) 16(11):2069–83. doi: 10.1080/15548627.2020.1714209 PMC7595620 31920150

[150] Kapralov AA Yang Q Dar HH Tyurina YY Anthonymuthu TS Kim R. Redox lipid reprogramming commands susceptibility of macrophages and microglia to ferroptotic death. Nat Chem Biol (2020) 16(3):278–90. doi: 10.1038/s41589-019-0462-8 PMC7233108 32080625

[151] Youssef LA Rebbaa A Pampou S Weisberg SP Stockwell BR Hod EA. Increased erythrophagocytosis induces ferroptosis in red pulp macrophages in a mouse model of transfusion. Blood J Am Soc Hematol (2018) 131(23):2581–93. doi: 10.1182/blood-2017-12-822619 PMC5992863 29666112

[152] Martinez J Malireddi R Lu Q Cunha LD Pelletier S Gingras S. Molecular characterization of LC3-associated phagocytosis reveals distinct roles for Rubicon, NOX2 and autophagy proteins. Nat Cell Biol (2015) 17(7):893–906. doi: 10.1038/ncb3192 26098576 PMC4612372

[153] Sanjuan MA Dillon CP Tait SW Moshiach S Dorsey F Connell S. Toll-like receptor signalling in macrophages links the autophagy pathway to phagocytosis. Nature (2007) 450(7173):1253–7. doi: 10.1038/nature06421 18097414

[154] Romao S Gasser N Becker AC Guhl B Bajagic M Vanoaica D. Autophagy proteins stabilize pathogen-containing phagosomes for prolonged MHC II antigen processing. J Cell Biol (2013) 203(5):757–66. doi: 10.1083/jcb.201308173 PMC3857489 24322427

[155] Wu W Bleecker E Moore W Busse WW Castro M Chung KF. Unsupervised phenotyping of severe asthma research program participants using expanded lung data. J Allergy Clin Immunol (2014) 133(5):1280–8. doi: 10.1016/j.jaci.2013.11.042 PMC4038417 24589344

[156] Anderson GP. Endotyping asthma: new insights into key pathogenic mechanisms in a complex, heterogeneous disease. Lancet (2008) 372(9643):1107–19. doi: 10.1016/S0140-6736(08)61452-X 18805339

[157] Woodruff PG Modrek B Choy DF Jia G Abbas AR Ellwanger A. T-Helper type 2–driven inflammation defines major subphenotypes of asthma. Am J Respir Crit Care Med (2009) 180(5):388–95. doi: 10.1164/rccm.200903-0392OC PMC2742757 19483109

[158] Puleston DJ Simon AK. Autophagy in the immune system. Immunology (2014) 141(1):1–8. doi: 10.1111/imm.12165 23991647 PMC3893844

[159] Kondylis V Van Nispen Tot Pannerden HE Van Dijk S Ten Broeke T Wubbolts R Geerts WJ. Endosome-mediated autophagy: an unconventional MIIC-driven autophagic pathway operational in dendritic cells. Autophagy (2013) 9(6):861–80. doi: 10.4161/auto.24111 PMC3672296 23481895

[160] Kearley J Barker JE Robinson DS Lloyd CM. Resolution of airway inflammation and hyperreactivity after *in vivo* transfer of CD4+ CD25+ regulatory T cells is interleukin 10 dependent. J Exp Med (2005) 202(11):1539–47. doi: 10.1084/jem.20051166 PMC1350743 16314435

[161] Hartl D Koller B Mehlhorn AT Reinhardt D Nicolai T Schendel DJ. Quantitative and functional impairment of pulmonary CD4+ CD25hi regulatory T cells in pediatric asthma. J Allergy Clin Immunol (2007) 119(5):1258–66. doi: 10.1016/j.jaci.2007.02.023 17412402

[162] Garg SK Yan Z Vitvitsky V Banerjee R. Differential dependence on cysteine from transsulfuration versus transport during T cell activation. Antioxidants Redox Signaling (2011) 15(1):39–47. doi: 10.1089/ars.2010.3496 20673163 PMC3110100

[163] Levring TB Hansen AK Nielsen BL Kongsbak M Von Essen MR Woetmann A. Activated human CD4+ T cells express transporters for both cysteine and cystine. Sci Rep (2012) 2(1):1–6. doi: 10.1038/srep00266 PMC3278673 22355778

[164] Pua HH Dzhagalov I Chuck M Mizushima N He Y-W. A critical role for the autophagy gene Atg5 in T cell survival and proliferation. J Exp Med (2007) 204(1):25–31. doi: 10.1084/jem.20061303 17190837 PMC2118420

[165] Jia W He Y-W. Temporal regulation of intracellular organelle homeostasis in T lymphocytes by autophagy. J Immunol (2011) 186(9):5313–22. doi: 10.4049/jimmunol.1002404 21421856

[166] Pua HH Guo J Komatsu M He Y-W. Autophagy is essential for mitochondrial clearance in mature T lymphocytes. J Immunol (2009) 182(7):4046–55. doi: 10.4049/jimmunol.0801143 19299702

[167] Willinger T Flavell RA. Canonical autophagy dependent on the class III phosphoinositide-3 kinase Vps34 is required for naive T-cell homeostasis. Proc Natl Acad Sci (2012) 109(22):8670–5. doi: 10.1073/pnas.1205305109 PMC3365213 22592798

[168] Parekh VV Wu L Boyd KL Williams JA Gaddy JA Olivares-Villagómez D. Impaired autophagy, defective T cell homeostasis, and a wasting syndrome in mice with a T cell–specific deletion of Vps34. J Immunol (2013) 190(10):5086–101. doi: 10.4049/jimmunol.1202071 PMC3646937 23596309

[169] Reed M Morris SH Jang S Mukherjee S Yue Z Lukacs NW. Autophagy-inducing protein beclin-1 in dendritic cells regulates CD4 T cell responses and disease severity during respiratory syncytial virus infection. J Immunol (2013) 191(5):2526–37. doi: 10.4049/jimmunol.1300477 PMC3811020 23894198

[170] Wang W Green M Choi JE Gijón M Kennedy PD Johnson JK. CD8+ T cells regulate tumour ferroptosis during cancer immunotherapy. Nature (2019) 569(7755):270–4. doi: 10.1038/s41586-019-1170-y PMC6533917 31043744

[171] Laberge S Wu L Olivenstein R Xu L-J Renzi PM Martin JG. Depletion of CD8+ T cells enhances pulmonary inflammation but not airway responsiveness after antigen challenge in rats. J Allergy Clin Immunol (1996) 98(3):617–27. doi: 10.1016/S0091-6749(96)70096-9 8828540

[172] Isogai S Jedrzkiewicz S Taha R Hamid Q Martin JG. Resident CD8+ T cells suppress CD4+ T cell–dependent late allergic airway responses. J Allergy Clin Immunol (2005) 115(3):521–6. doi: 10.1016/j.jaci.2004.11.036 15753899

[173] Tsuchiya K Isogai S Tamaoka M Inase N Akashi T Martin JG. Depletion of CD8+ T cells enhances airway remodelling in a rodent model of asthma. Immunology (2009) 126(1):45–54. doi: 10.1111/j.1365-2567.2008.02876.x 18564065 PMC2632694

[174] Mahmutovic Persson I Menzel M Ramu S Cerps S Akbarshahi H Uller L. IL-1β mediates lung neutrophilia and IL-33 expression in a mouse model of viral-induced asthma exacerbation. Respir Res (2018) 19(1):1–10. doi: 10.1186/s12931-018-0725-z 29361942 PMC5781288

[175] Xu C Sun S Johnson T Qi R Zhang S Zhang J. The glutathione peroxidase Gpx4 prevents lipid peroxidation and ferroptosis to sustain treg cell activation and suppression of antitumor immunity. Cell Rep (2021) 35(11):109235. doi: 10.1016/j.celrep.2021.109235 34133924

[176] Xiong A Duan L Chen J Fan Z Zheng F Tan Z. Flt3L combined with rapamycin promotes cardiac allograft tolerance by inducing regulatory dendritic cells and allograft autophagy in mice. Cell Reports (2012) 35(11). doi: 10.1371/journal.pone.0046230 PMC3462742 23056267

[177] Hubbard-Lucey VM Shono Y Maurer K West ML Singer NV Ziegler CG. Autophagy gene Atg16L1 prevents lethal T cell alloreactivity mediated by dendritic cells. Immunity (2014) 41(4):579–91. doi: 10.1016/j.immuni.2014.09.011 PMC4237219 25308334

[178] Kim HY DeKruyff RH Umetsu DT. The many paths to asthma: phenotype shaped by innate and adaptive immunity. Nat Immunol (2010) 11(7):577–84. doi: 10.1038/ni.1892 PMC3114595 20562844

[179] De Vooght V Carlier V Devos FC Haenen S Verbeken E Nemery B. B-lymphocytes as key players in chemical-induced asthma. PloS One (2013) 8(12):e83228. doi: 10.1371/journal.pone.0083228 24349469 PMC3862726

[180] Miller BC Zhao Z Stephenson LM Cadwell K Pua HH Lee HK. The autophagy gene ATG5 plays an essential role in b lymphocyte development. Autophagy (2008) 4(3):309–14. doi: 10.4161/auto.5474 18188005

[181] Muri J Thut H Bornkamm GW Kopf M. B1 and marginal zone b cells but not follicular B2 cells require Gpx4 to prevent lipid peroxidation and ferroptosis. Cell Rep (2019) 29(9):2731–44. e4. doi: 10.1016/j.celrep.2019.10.070 31775041

[182] Pengo N Scolari M Oliva L Milan E Mainoldi F Raimondi A. Plasma cells require autophagy for sustainable immunoglobulin production. Nat Immunol (2013) 14(3):298–305. doi: 10.1038/ni.2524 23354484

[183] Yao Y Chen Z Zhang H Chen C Zeng M Yunis J. Selenium–GPX4 axis protects follicular helper T cells from ferroptosis. Nat Immunol (2021) 22(9):1127–39. doi: 10.1038/s41590-021-00996-0 34413521

[184] Xia F Deng C Jiang Y Qu Y Deng J Cai Z. IL4 (interleukin 4) induces autophagy in b cells leading to exacerbated asthma. Autophagy (2018) 14(3):450–64. doi: 10.1080/15548627.2017.1421884 PMC5915013 29297752

[185] Wang D Xie N Gao W Kang R Tang D. The ferroptosis inducer erastin promotes proliferation and differentiation in human peripheral blood mononuclear cells. Biochem Biophys Res Commun (2018) 503(3):1689–95. doi: 10.1016/j.bbrc.2018.07.100 PMC6179365 30049441

[186] Liu J-N Suh D-H Trinh HKT Chwae Y-J Park H-S Shin YS. The role of autophagy in allergic inflammation: a new target for severe asthma. Exp Mol Med (2016) 48(7):e243–e. doi: 10.1038/emm.2016.38 PMC4973311 27364893

[187] Wu Y Chen H Xuan N Zhou L Wu Y Zhu C. Induction of ferroptosis-like cell death of eosinophils exerts synergistic effects with glucocorticoids in allergic airway inflammation. Thorax (2020) 75(11):918–27. doi: 10.1136/thoraxjnl-2020-214764 32759385

[188] Bianchi M Hakkim A Brinkmann V Siler U Seger RA Zychlinsky A. Restoration of NET formation by gene therapy in CGD controls aspergillosis. Blood J Am Soc Hematol (2009) 114(13):2619–22. doi: 10.1182/blood-2009-05-221606 PMC2756123 19541821

[189] Li P Li M Lindberg MR Kennett MJ Xiong N Wang Y. PAD4 is essential for antibacterial innate immunity mediated by neutrophil extracellular traps. J Exp Med (2010) 207(9):1853–62. doi: 10.1084/jem.20100239 PMC2931169 20733033

[190] Xu F Zhang C Zou Z Fan EK Chen L Li Y. Aging-related Atg5 defect impairs neutrophil extracellular traps formation. Immunol (2017) 151(4):417–32. doi: 10.1111/imm.12740 PMC5506403 28375544

[191] Kambas K Mitroulis I Ritis K. The emerging role of neutrophils in thrombosis–the journey of TF through NETs. Front Immunol (2012) 3:385. doi: 10.3389/fimmu.2012.00385 23264778 PMC3524512

[192] Yotsumoto S Muroi Y Chiba T Ohmura R Yoneyama M Magarisawa M. Hyperoxidation of ether-linked phospholipids accelerates neutrophil extracellular trap formation. Sci Rep (2017) 7(1):1–18. doi: 10.1038/s41598-017-15668-z 29167447 PMC5700140

[193] Bhattacharya A Wei Q Shin JN Fattah EA Bonilla DL Xiang Q. Autophagy is required for neutrophil-mediated inflammation. Cell Rep (2015) 12(11):1731–9. doi: 10.1016/j.celrep.2015.08.019 26344765

[194] Li W Feng G Gauthier JM Lokshina I Higashikubo R Evans S. Ferroptotic cell death and TLR4/Trif signaling initiate neutrophil recruitment after heart transplantation. J Clin Invest (2019) 129(6):2293–304. doi: 10.1172/JCI126428 PMC6546457 30830879

[195] Ushio H Ueno T Kojima Y Komatsu M Tanaka S Yamamoto A. Crucial role for autophagy in degranulation of mast cells. J Allergy Clin Immunol (2011) 127(5):1267–76. e6. doi: 10.1016/j.jaci.2010.12.1078 21333342

[196] Yoo S-E Chen L Na R Liu Y Rios C Van Remmen H. Gpx4 ablation in adult mice results in a lethal phenotype accompanied by neuronal loss in brain. Free Radical Biol Med (2012) 52(9):1820–7. doi: 10.1016/j.freeradbiomed.2012.02.043 PMC3341497 22401858

[197] McAlinden KD Deshpande DA Ghavami S Xenaki D Sohal SS Oliver BG. Autophagy activation in asthma airways remodeling. Am J Respir Cell Mol Biol (2019) 60(5):541–53. doi: 10.1165/rcmb.2018-0169OC PMC6503620 30383396

[198] Alizadeh J Glogowska A Thliveris J Kalantari F Shojaei S Hombach-Klonisch S. Autophagy modulates transforming growth factor beta 1 induced epithelial to mesenchymal transition in non-small cell lung cancer cells. Biochim Biophys Acta (BBA)-Molecular Cell Res (2018) 1865(5):749–68. doi: 10.1016/j.bbamcr.2018.02.007 29481833

[199] Alizadeh J Shojaei S Sepanjnia A Hashemi M Eftekharpour E Ghavami S. Simultaneous detection of autophagy and epithelial to mesenchymal transition in the non-small cell lung cancer cells. Autophagy Differentiation Tissue Maintenance (2017) 1854:87–103. doi: 10.1007/7651_2017_84 29101677

[200] Wu Y Zhang S Gong X Tam S Xiao D Liu S. The epigenetic regulators and metabolic changes in ferroptosis-associated cancer progression. Mol cancer (2020) 19(1):1–17. doi: 10.1186/s12943-020-01157-x 32103754 PMC7045519

[201] Banerjee P Balraj P Ambhore NS Wicher SA Britt RD Pabelick CM. Network and co-expression analysis of airway smooth muscle cell transcriptome delineates potential gene signatures in asthma. Sci Rep (2021) 11(1):1–16. doi: 10.1038/s41598-021-93845-x 34257337 PMC8277837

